# Systematic review of risk prediction models for sepsis-associated brain dysfunction

**DOI:** 10.3389/fneur.2026.1653460

**Published:** 2026-02-27

**Authors:** Wen Shen, Ting Li, Yun Wang, Ping Jia, Xia Zeng

**Affiliations:** 1 School of Nursing, Chengdu University of Traditional Chinese Medicine, Chengdu, China; 2 Department of Intensive Care Unit, Sichuan Provincial People's Hospital, School of Medicine, University of Electronic Science and Technology of China, Chengdu, China; 3 Emergency Intensive Care Unit (EICU), Sichuan Academy of Medical Sciences, Sichuan Provincial People's Hospital (Affiliated Hospital of University of Electronic Science and Technology of China), Chengdu, China

**Keywords:** risk prediction model, sepsis, sepsis-associated delirium, sepsis-associated encephalopathy, systematic review

## Abstract

**Objective:**

To systematically review the outcome constructs, modeling characteristics, and methodological quality of existing sepsis-associated brain dysfunction (SABD) risk prediction models, with the aim of explaining why current models are difficult to reproduce or translate into practice, and of proposing standardized directions for future research.

**Methods:**

A systematic review was conducted by searching CNKI, Wanfang, VIP, SinoMed, PubMed, CINAHL, Cochrane Library, Embase, and Web of Science from database inception to April 2025. Studies developing or validating SABD risk prediction models were included, with outcomes defined as sepsis-associated encephalopathy (SAE) or sepsis-associated delirium (SAD). Model characteristics were extracted according to the CHARMS checklist, and methodological quality was assessed using the Prediction model Risk of Bias Assessment Tool (PROBAST).

**Results:**

Twelve studies involving 24 risk prediction models were included, of which four studies evaluated SAD as the outcome and eight evaluated SAE. Substantial heterogeneity was observed in outcome definitions, modeling strategies, and variable selection approaches. Calibration was reported in 10 studies, internal validation in nine studies, and both internal and external validation in one study. According to PROBAST, three studies had high applicability concerns and nine had low applicability concerns. All included studies were assessed as having a high risk of bias, predominantly in the analysis domain.

**Conclusion:**

Current risk prediction modeling studies for SAD and SAE remain exploratory, and high risk of bias together with insufficient validation limits their reliable clinical translation. Future research should adhere to the PROBAST and TRIPOD guidelines, conduct multicenter prospective studies, and standardize modeling and validation procedures.

**Systematic review registration:**

https://www.crd.york.ac.uk/, identifier CRD420251014680.

## 1 Introduction

Sepsis is defined as life-threatening organ dysfunction caused by a dysregulated host response to infection. Globally, approximately 48 million people develop sepsis each year and about 11 million die from it, making sepsis a major global health challenge (1, 2). Sepsis-associated brain dysfunction (SABD) refers to acute brain dysfunction occurring during the onset and progression of sepsis, typically manifested as altered consciousness and mental status, impaired attention and orientation, cognitive decline, and behavioral abnormalities (3). Evidence suggests that sepsis patients with brain dysfunction generally have poorer outcomes, including a higher short-term risk of death, longer hospital stays, and greater healthcare resource utilization, and some patients continue to experience persistent neurocognitive impairment after discharge (4, 5). Therefore, early identification of high-risk patients and implementation of targeted monitoring and interventions are key components of critical care management.

However, the underlying mechanisms of SABD have not been fully elucidated and may involve multiple pathways, including systemic inflammatory responses, blood–brain barrier dysfunction, disturbances of cerebral microcirculation, and alterations in neurotransmission and metabolic processes (6). Because of the lack of specific biomarkers and standardized diagnostic criteria, clinical identification still relies primarily on symptom presentation and the use of operationalized assessment tools. In the literature, brain dysfunction during sepsis has been described using different terms. Many studies refer to it as sepsis-associated encephalopathy (SAE) (7). Given the overlap between its clinical features and delirium in critically ill patients, and because delirium can be assessed in a relatively standardized bedside manner using tools such as the CAM-ICU, some studies have instead used sepsis-associated delirium (SAD) as the outcome (8, 9). However, inconsistent terminology and diagnostic ambiguity further increase the difficulty of clinical recognition and of conducting high-quality research.

In recent years, studies on risk prediction models for SABD have increased. However, substantial heterogeneity in outcome definitions, assessment time windows, predictor handling, and validation strategies across studies makes model results difficult to interpret reliably and limits their clinical translation. In this context, we conducted a systematic review using SABD in a broad sense as the conceptual framework and restricted the included outcomes to two relatively operational and widely used outcome measurement approaches in the literature, namely SAE and SAD. Our aim was to systematically summarize the outcome constructs and methodological characteristics of existing models, in order to help explain why their clinical translatability remains limited and to inform the standardized design of future research.

## 2 Methods

### 2.1 Literature search strategy

A comprehensive search was performed in major Chinese and English databases, including CNKI, Wanfang, VIP, SinoMed, PubMed, CINAHL, the Cochrane Library, Embase, and Web of Science, from database inception to April 2025. We also screened the reference lists of included studies to identify additional eligible publications. The literature search included the following search terms: “Sepsis-Associated Encephalopathy,” “SAE,” “SAD,” “Sepsis Associated Encephalopathy,” “Septic encephalopathy,” “Sepsis-Associated Delirium,” “Sepsis Associated Deliriums,” “Prediction model,” “Prediction*,” “Risk Prediction,” “Nomogram,” “Machine learning.” The search procedure is shown in Supplementary Table S1.

### 2.2 Literature inclusion and exclusion criteria

#### 2.2.1 Inclusion criteria

Based on the PICOS principle, the inclusion criteria were as follows: (1) Participants: adults aged ≥18 years with sepsis defined according to Sepsis-3 or with a clearly stated sepsis ascertainment method in the study; (2) Content: studies that developed or validated multivariable risk prediction models for SABD using traditional regression approaches or machine learning algorithms; (3) Outcomes: the target outcome was explicitly defined as SAE or SAD, and the study provided a clear definition or diagnostic criteria for the outcome; (4) Study design: prospective or retrospective studies.

#### 2.2.2 Exclusion criteria

(1) Studies only identifying risk factors without developing prediction models; (2) Duplicate reports of the same prediction model or study cohort (the most complete or latest version was retained); (3) Full text unavailable or insufficient information to extract key model characteristics and performance; (4) Non-original data (meta-analyses, guidelines, case reports, etc.).

### 2.3 Literature screening and data extraction

Two professionals independently conducted literature screening, including initial duplicate screening and screening based on titles and abstracts. For the qualified initial screening literature, full texts were downloaded for re-screening to determine the final inclusion. In case of disagreement, a third researcher would be involved in the decision-making. Data extraction was based on the Critical Appraisal and Data Extraction for Systematic Reviews of Prediction Modeling Studies (CHARMS) checklist (10), covering basic research information, sample characteristics, construction methods of prediction models, performance indicators, validation methods, and presentation methods of models, to ensure the comprehensiveness and consistency of the systematic review.

### 2.4 Literature quality evaluation

In this systematic review of prediction model studies, we used the PROBAST tool (11) (Prediction Model Risk of Bias Assessment Tool) to assess risk of bias and applicability. PROBAST evaluates four domains for risk of bias (participants, predictors, outcome, and analysis) and three domains for applicability concerns (participants, predictors, and outcome). The overall risk of bias was judged as “high” if at least one domain was rated as high risk; it was judged as “low” only if all domains were rated as low risk; otherwise, the overall judgment was “unclear.” Two investigators independently performed the assessments, and any discrepancies were resolved through discussion with a third reviewer.

## 3 Results

### 3.1 Results of the literature search

**Figure 1:**
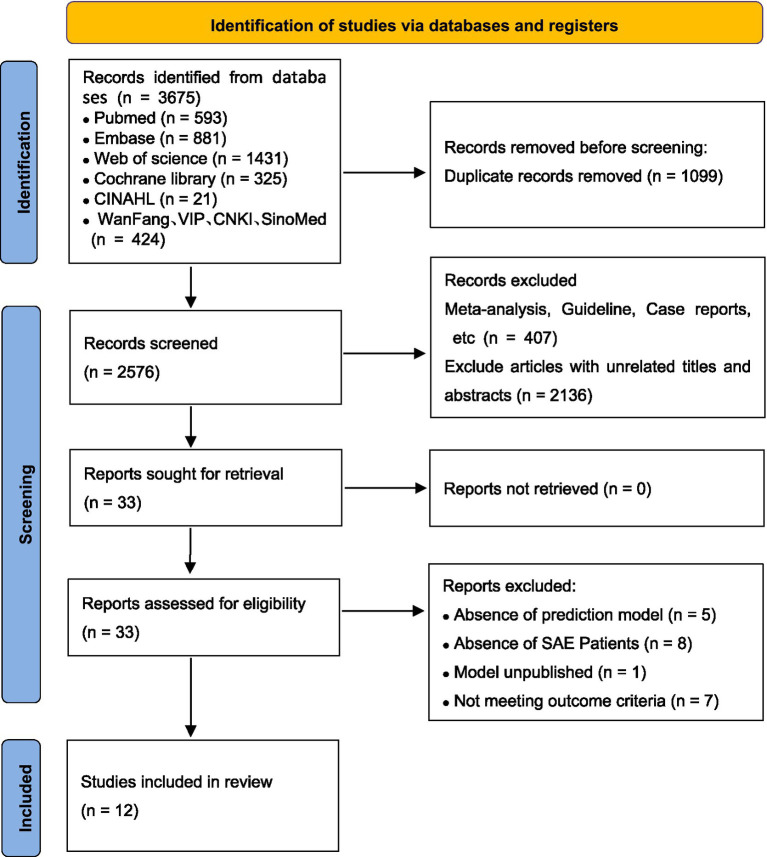
Screening diagram of literature process.

In the initial database search, 3,675 articles were found, with 2,576 selected for primary screening after removing duplicates. During preliminary evaluation, articles unrelated to the research topic were excluded, leaving 33 for full-text review. Of these, 21 were excluded for reasons such as lacking specific predictors, focusing solely on sepsis, not establishing predictive models, or primarily studying mortality. Ultimately, 12 articles were included in the systematic review (2, 12–22). The study selection flowchart is shown in Figure 1.

### 3.2 Basic characteristics of included literature

A total of 12 studies published between 2021 and 2025 were included (2, 12–22), of which 7 were published in English (2, 12, 15–19). Regarding data sources, 6 studies were based on the MIMIC-III or MIMIC-IV databases and mainly included patients from the United States (2, 12, 15, 16, 18, 19), whereas the other 6 studies used single-center datasets from hospitals in China (13, 14, 17, 20–22). In terms of study design, 11 studies were single-center retrospective cohorts (2, 12–14, 16–22), and only 1 was a prospective study (17). The included studies used either SAD or SAE as the outcome, and the study populations primarily included older patients. Among studies using SAD as the outcome, sample sizes ranged from 308 to 14,620 patients, with 103–5,390 events and incidence rates of approximately 29.92–36.87%. Among studies using SAE as the outcome, sample sizes ranged from 67 to 22,361 patients, with 32–8,290 events and incidence rates of approximately 15.16–62.13%. The basic characteristics of the literature are shown in Table 1.

**Table 1:** Basic characteristics of included studies, stratified by outcome definition (SAD vs. SAE).

Author	Year	Source	Patients	Study design	Model type	Age (MD/MV)	Sample (MD/MV)		
							Cases	Events	Incidence
Gu Q (12)	2023	MIMIC-III	SAD	①	MD + IV	68.22 ± 16.55	642	228	35.51%
Zhang Y (2)	2023	MIMIC-IV eICU-CRD	SAD	①	MD + IV + EV	68.24 ± 16.59	14,620	5,390	36.87%
Li (13)	2024	Chinese	SAD	①	MD	71.41 ± 6.49	308	103	33.44%
Yang (14)	2024	Chinese	SAD	①	MD	-	381	114	29.92%
Jin J (15)	2024	MIMIC-IV	SAE	①	MD + IV	66.30 ± 14.83	4,476	2,781	62.13%
Zhao L (16)	2021	MIMIC-III	SAE	①	MD + IV	68.88 ± 16.69	2028	841	41.50%
						69.31 ± 19.04	507	214	42.20%
Mei J (17)	2024	Chinese	SAE	②	MD	58.17 ± 10.02	67	32	47.76%
Lu X (18)	2022	MIMIC-IV	SAE	①	MD + IV	67.26 ± 16.38	8,935	4,684	52.42%
Zhao Q (19)	2023	MIMIC-IV	SAE	①	MD + IV	77.25 ± 8.05	22,361	8,290	37.07%
Wang (20)	2023	Chinese	SAE	①	MD + IV	56.20 ± 16.49 59.65 ± 18.62	640	97	15.16%
Zhou (21)	2023	Chinese	SAE	①	MD	-	213	84	39.44%
Zhang (22)	2024	Chinese	SAE	①	MD	59.40 ± 17.68	130	52	40.00%

MIMIC-III, Medical Information Mart for Intensive Care III; MIMIC-IV, Medical Information Mart for Intensive Care IV; eICU-CRD, eICU Collaborative Research Database; Chinese, study-specific Chinese clinical cohort; SAD, sepsis-associated delirium; SAE, sepsis-associated encephalopathy; MD, model development; IV, internal validation; EV, external validation; ① retrospective study; ② prospective study; “-,” not reported.

### 3.3 Definition and criteria for outcomes

The included studies employed two operational outcome measurement approaches, and we report their results in a stratified manner accordingly. Four studies used SAD as the outcome, typically assessed within a predefined time window using delirium assessment instruments such as the CAM-ICU (2, 12–14). The other eight studies used SAE as the outcome. In these studies, SAE was most commonly defined by a decreased GCS score (e.g., GCS < 15 or ≤ 14) together with documentation of delirium (based on either scale assessments or medical record notes), with non-uniform assessment time points across studies (15–22). We therefore describe and present findings for SAD and SAE separately, reflecting the outcome definitions and assessment time points reported in the included studies. Outcome definitions and ascertainment criteria are shown in Table 2.

**Table 2:** Outcome definitions and ascertainment criteria of included studies, stratified by outcome type (SAD vs. SAE).

Author	Outcome term	Diagnostic basis	Definition	Assessment	Time of assessment
Gu Q (12)	SAD	Sepsis-3	Suspected or confirmed infection with a SOFA score ≥ 2 points	SOFA score; CAM-ICU	Within 24 h
Zhang Y (2)	SAD	Sepsis-3	Suspected or confirmed infection with a SOFA score ≥ 2 points	SOFA score; CAM-ICU	Within 24 h
Li (13)	SAD	Sepsis-3	Suspected or confirmed infection with a SOFA score ≥ 2 points	SOFA score; CAM-ICU	Within 24 h
Yang (14)	SAD	Sepsis-3	Suspected or confirmed infection with a SOFA score ≥ 2 points	SOFA score; CAM-ICU	Within 24 h
Jin J (15)	SAE	Sepsis-3	GCS score < 15 points or documentation of delirium in medical records, including inattention, disorientation, altered thinking, psychomotor retardation and/or agitation	GCS; delirium	-
Zhao L (16)	SAE	Sepsis-3	GCS score < 15 points or documentation of delirium in medical records, including inattention, disorientation, altered thinking, psychomotor retardation and/or agitation	GCS; delirium	Within 24 h
Mei J (17)	SAE	Sepsis-3	GCS score < 15 points, or a diagnosis of delirium based on the CAM-ICU checklist.	GCS; CAM-ICU	Within 24 h
Lu X (18)	SAE	Sepsis-3	GCS ≤ 14 or delirium	GCS; delirium	-
Zhao Q (19)	SAE	Sepsis-3	GCS<15 or delirium	GCS; delirium	Within 24 h
Wang (20)	SAE	Sepsis-3	GCS score < 15 points or documentation of delirium in medical records, including inattention, disorientation, altered thinking, psychomotor retardation and/or agitation	GCS; delirium	Within 24 h
Zhou (21)	SAE	Sepsis-3	Suspected or confirmed infection with a SOFA score ≥ 2 points	SOFA score; CAM-ICU	Within 24 h
Zhang (22)	SAE	Sepsis-3	-	-	-

Sepsis-3, The Third International Consensus Definitions for Sepsis and Septic Shock, defines sepsis as suspected or documented infection accompanied by an acute increase in the Sequential Organ Failure Assessment (SOFA) score of ≥2 points; GCS, Glasgow Coma Scale; CAM-ICU, Confusion Assessment Method for the Intensive Care Unit, composed of four core features: (1) acute onset or fluctuating mental status; (2) inattention; (3) disorganized thinking; and (4) altered level of consciousness. A positive CAM-ICU result (i.e., delirium) is defined as the presence of Features 1 and 2 plus either Feature 3 or 4; MMSE, Mini-Mental State Examination; MoCA, Montreal Cognitive Assessment; “–”, not reported.

### 3.4 Basic characteristics of the prediction model

**Figure 2:**
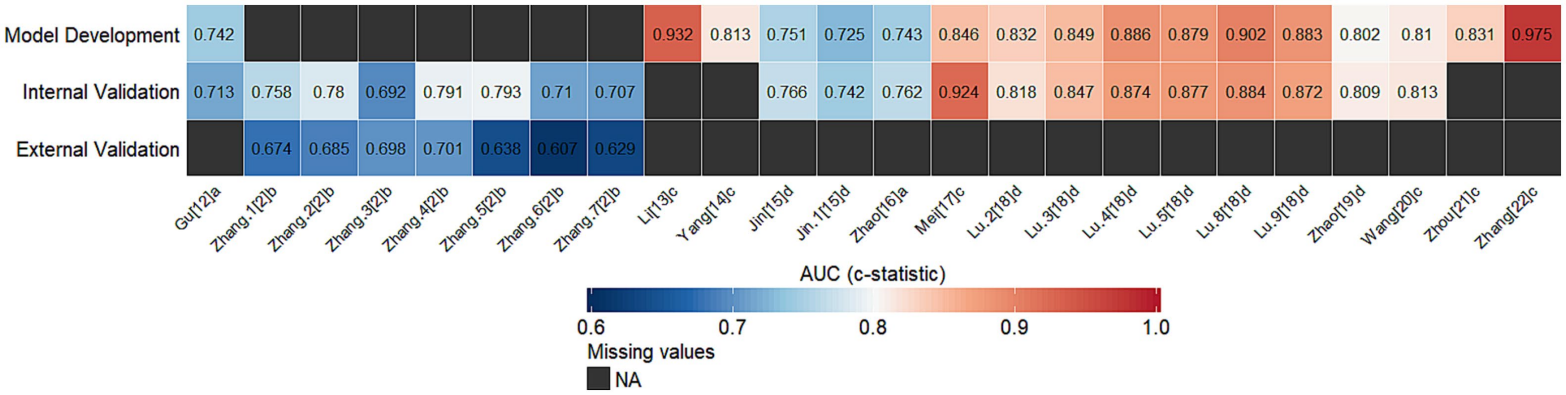
Reported AUC (c-statistic) values of included SABD risk prediction models (SAD vs. SAE). a, MIMIC-III; b, MIMIC-IV (internal validation) and eICU-CRD (external validation); c, study-specific Chinese clinical cohorts; d, MIMIC-IV; 1, LR; 2, SVM; 3, DT; 4, RF; 5, XGBoost; 6, NB; 7, KNN; 8, GBM; 9, MLP; 10, LGBM; 11, GBTD.

Across the 12 included studies, a total of 24 risk prediction models were reported. Four studies developed risk prediction models with SAD as the outcome (2, 12–14). Regarding variable selection, one study used LASSO for predictor selection (2), whereas the other three first conducted univariable analyses and then entered selected variables into multivariable models (12–14). Model development was mainly based on logistic regression, and one study additionally applied machine learning methods and developed seven models (2). Regarding variable handling, one study transformed continuous variables into categorical variables (14). For missing data handling, one study used Multiple Imputation (2), while the others did not report specific handling methods. For model calibration assessment, all four studies primarily relied on the Hosmer-Lemeshow test or calibration curves (2, 12–14); two studies additionally reported decision curve analysis across different decision thresholds (2, 12). Regarding model validation, two studies performed internal validation using a split-sample approach (2, 12). Among them, one study combined internal validation with external validation (2). In addition, one study conducted internal validation using bootstrap resampling during model development (13), while another study did not report any validation procedures. Regarding model presentation, two studies displayed their prediction models as nomograms (12, 13), one provided a risk scoring formula (14), and the other did not provide a clear presentation format. Across the included SAD models, the reported AUC (c-statistic) values are presented in Figure 2 and Table 3. When multiple validation AUC (c-statistic) estimates were available within a single study, a prespecified hierarchical extraction rule was applied, prioritizing external validation, followed by temporal or geographic validation, internal resampling methods, split-sample validation, and apparent performance if no other estimates were available.

**Table 3:** Basic characteristics of included risk prediction models, stratified by outcome type (SAD vs. SAE).

Author	Data source/cohort	Variable selection	Modeling methods	Variable handling	Model performance		Model validation	EPV	Missing data	Model presentation
					AUC (c-statistic) (MD/IV/EV)	Calibration				
Gu Q (12)	MIMIC-III/MD + IV	UA, MA	LR	a	0.742/0.713/-	H-L Calibration curves DCA	Internal validation (split-sample)	57	-	N
Zhang Y (2)	MIMIC-IV/MD + IV eICU-CRD/EV	Lasso, UA	LR SVM DT RF XGBoost NB KNN	a	-/0.758/0.674 -/0.780/0.685 -/0.692/0.698 -/0.791/0.701 -/0.793/0.638 -/0.710/0.607 -/0.707/0.629	Calibration curves DCA	Internal validation (split-sample)/External validation	-	MI	-
Li (13)	Chinese/MD	UA, MA	LR	a	0.932/-/-	Calibration curves	Internal validation (bootstrap)	10.3	-	N
Yang (14)	Chinese/MD	UA, MA	LR	b	0.813/-/-	H-L Calibration curves	-	19	-	F
Jin J (15)	MIMIC-IV/MD + IV	Lasso, UA, MA	LR	a	0.751/0.766/- 0.725/0.742/-	H-L Calibration curves	Internal validation (split-sample)	309	MI	N
Zhao L (16)	MIMIC-III/MD + IV	Lasso, UA, MA	LR	a	0.743/0.762/-	Calibration curves	Internal validation (split-sample)	93.44/23.78	-	N
Mei J (17)	Chinese/MD	UA, MA	LR	a	0.846/0.924/-	H-L Calibration curves DCA	Internal validation (bootstrap)	10.67	-	N
Lu X (18)	MIMIC-IV/MD + IV	UA	SVM DT RF XGBoost GBM GBDT	a	0.832/0.818/- 0.849/0.847/- 0.886/0.874/- 0.902/0.884/- 0.879/0.877/- 0.883/0.872/-	-	Internal validation (split-sample)	-	MI	-
Zhao Q (19)	MIMIC-IV/MD + IV	UA, MA	LR	a	0.802/0.809/-	Calibration curvesDCA	Internal validation (split-sample, 10-FCV)	1,658	Excluded	N
Wang (20)	Chinese/MD + IV	UA, MA	LR	a	0.810/0.813/-	H-L Calibration curves	Internal validation (temporal validation)	19.4	-	N
Zhou (21)	Chinese/MD	UA, MA	LR	b	0.831/-/-	H-L Calibration curves	Internal validation (bootstrap)	12	-	N
Zhang (22)	Chinese/MD	UA, MA	LR	a	0.975/-/-	-	-	8.67	-	-

MD, model development; IV, internal validation; EV, external validation; Chinese, study-specific Chinese clinical cohort; EPV, events per variable; UA, univariate analysis; MA, multivariate analysis; LASSO, least absolute shrinkage and selection operator; LR, logistic regression; SVM, support vector machine; DT, decision tree; RF, random forest; XGBoost, extreme gradient boosting; NB, naive Bayes; KNN, k-nearest neighbors; GBM, gradient boosting machine; GBDT, gradient boosting decision trees; a, continuous variable; b, categorical variable; AUC, Area Under the Receiver Operating Characteristic Curve; c-statistic, concordance statistic; H-L, Hosmer-Lemeshow test; DCA, decision curve analysis; 10-FCV, 10-fold cross-validation; MI, multiple imputation; SI, single imputation; N, nomogram model; F, formula for the risk score derived from the partial regression coefficients of each factor; “–”, not reported.

Eight studies developed risk prediction models with SAE as the outcome (15–22). Regarding variable selection, two studies used LASSO (15, 16), one relied solely on univariable analysis (18), and the remaining five first performed univariable analyses and then entered selected variables into multivariable models (17, 19–22). Model development was likewise mainly based on logistic regression, and one study additionally applied machine learning methods and developed six models (18). Regarding variable handling, one study transformed continuous variables into categorical variables (21). For missing data handling, two studies used Multiple Imputation (15, 18), one study directly excluded cases with missing data (19), and the remaining studies did not report specific handling methods. For model calibration assessment, six studies primarily relied on the Hosmer-Lemeshow test or calibration curves (15–17, 19–21); two studies additionally reported decision curve analysis across different decision thresholds (17, 19); and two studies reported only AUC (c-statistic), without providing a more comprehensive assessment of calibration or clinical utility (18, 22). Regarding model validation, four studies (15, 16, 18, 19) performed internal validation using a random split-sample approach. Two additional studies conducted internal validation during model development (17, 21). Furthermore, one study performed temporal validation based on different time periods within the same cohort (20), while the remaining study reported only model development without any validation. Regarding model presentation, six studies displayed their prediction models as nomograms (15–17, 19–21), whereas the others did not provide complete model parameters. Across the included SAE models, the reported AUC (c-statistic) values are presented in Figure 2 and Table 3. The same prespecified hierarchical extraction rule was applied. The modeling characteristics of the included studies are shown in Table 3.

### 3.5 Inclusion of predictive factors

**Figure 3:**
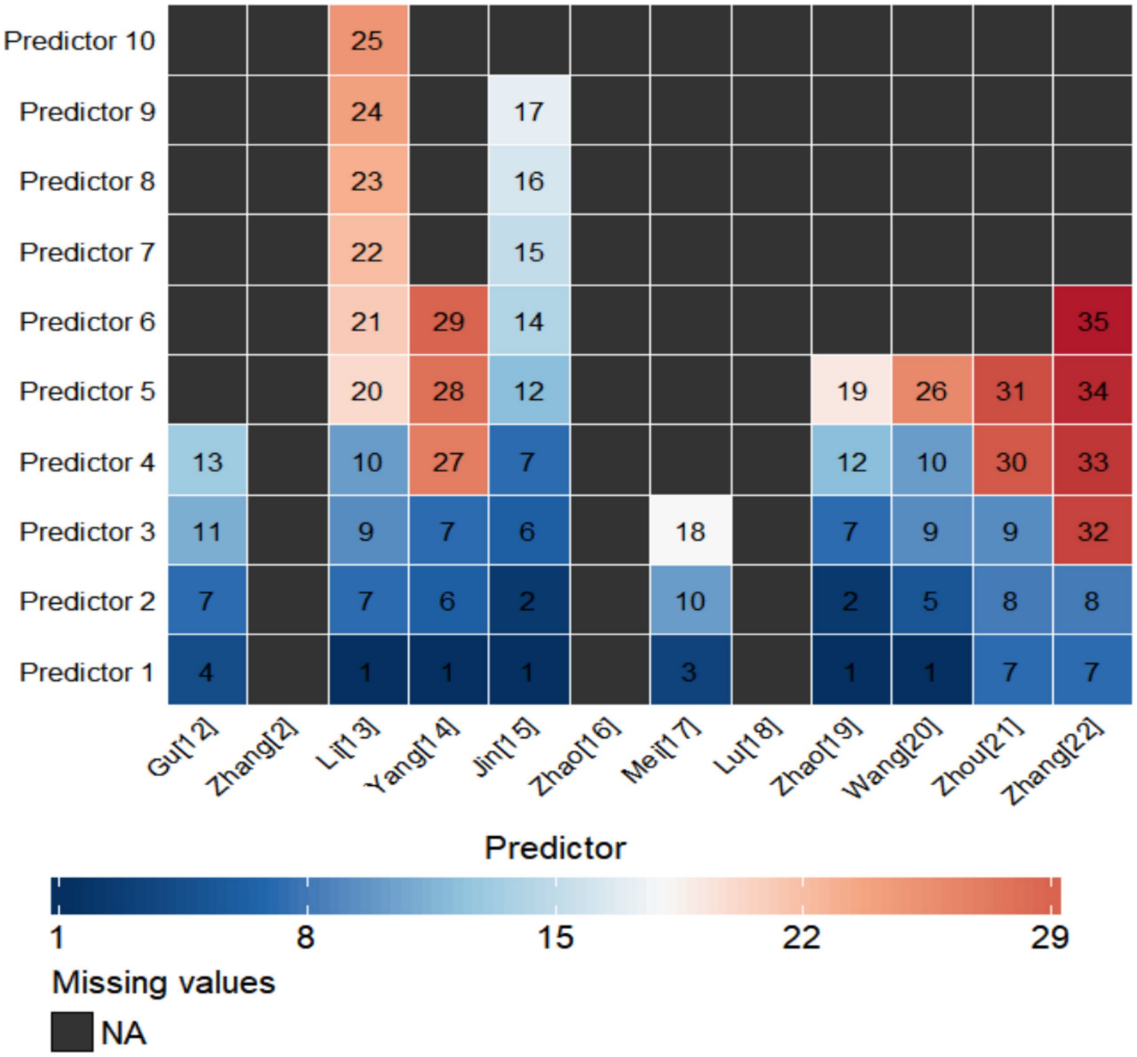
Predictors of included SABD risk prediction models (SAD vs. SAE). 1, Age; 2, Temperature; 3, Pulsatility Index, PI; 4, Mechanical Ventilation, MV; 5, Vasopressors; 6, Midazolam; 7, SOFA; 8, APACHE II; 9, Albumin, ALB; 10, S100 Calcium-Binding Protein B, S100B; 11, Lactate; 12, Sodium; 13, Phosphate; 14, BMI; 15, Mean Arterial Pressure, MAP; 16, Platelet, PLT; 17, Gender; 18, CCT; 19, Heart Rate; 20, Hyperglycemia; 21, Septic Shock; 22, Hypercapnia; 23, Sedatives Use; 24, Interleukin-6, IL-6; 25, Homocysteine, Hcy; 26, Partial Pressure of Oxygen in Arterial Blood, PaO2; 27, Coagulopathy; 28, Urea; 29, Metabolic Acidosis; 30, Regional Cerebral Oxygen Saturation, rScO₂; 31, Alanine Aminotransferase, ALT; 32, Pulmonary Infection; 33, Hemoglobin; 34, Charlson Comorbidity Index, CCI; 35, Gamma-Glutamyl Transferase, GGT.

From the 12 included studies, we extracted 35 candidate predictors used in the reported models. These predictors were grouped into five categories: demographic characteristics, disease severity scores, therapeutic interventions, physiological and laboratory measures, and specialized monitoring indicators. Several predictors appeared in more than one model. In models with SAD as the outcome, predictors that appeared in more than one model included age (*n* = 2) (13, 14) and SOFA score (*n* = 3) (12–14) (see Figure 3). In models with SAE as the outcome, predictors that appeared in more than one model included age (*n* = 3) (15, 19, 20), SOFA score (*n* = 4) (15, 19, 21, 22), APACHE II score (*n* = 2) (21, 22), body temperature (*n* = 2) (15, 19), and serum sodium (*n* = 2) (15, 19) (see Figure 3).

### 3.6 Results of quality assessment

Two reviewers used PROBAST to assess the risk of bias and applicability of the 12 included studies (four with SAD as the outcome and eight with SAE as the outcome). Because the overall risk of bias patterns across the four PROBAST domains were comparable between SAD- and SAE-based models, the PROBAST results were summarized jointly. In the participants domain, 11 studies were retrospective and relied on existing case data and were rated as high risk of bias (2, 12–16, 18–22). In the predictors domain, 11 studies did not clearly report whether predictor assessment was conducted independently of outcome determination or whether blinding was implemented and were rated as having an unclear risk of bias (2, 12–16, 18–22). In the outcome domain, only one study prespecified outcome criteria and was assessed as having a low risk of bias (15). Another study (17) was prospective, with outcomes not yet occurring at predictor collection, and blinding was rated as ensured. This study was assessed as having a low risk of bias in the participants, predictors, and outcome domains.

In the analysis domain, four studies met the EPV ≥ 20 criterion (12, 15, 16, 19), whereas eight studies (2, 13, 14, 17, 18, 20–22) had an EPV < 20 and did not report subsequent adjustment of model parameters and were rated as high risk of bias. Regarding variable selection, one study selected predictors based solely on univariable analysis and was rated as high risk of bias (18). Regarding variable handling, two studies categorized continuous variables and were rated as high risk of bias (14, 21). Regarding data handling, eight studies did not report how missing data were handled (12–14, 16, 17, 19–22), and one study excluded cases with missing values and was rated as high risk of bias (19). Regarding model calibration, all included studies reported discrimination, but two studies did not report calibration (18, 22). Regarding model validation, two studies reported model development only without model validation (14, 22).

Regarding applicability, three studies were judged to have a high risk of applicability concerns (15, 18, 22), while nine studies were assessed as having low applicability concerns (2, 12–14, 16, 17, 19–21). In the participants domain, the study by Zhang (22) did not fully report the outcome definition and was judged to have high applicability concerns. In the Predictors domain, three studies did not clearly report the timing of outcome assessment and were judged to have high applicability concerns (15, 18, 22). In the outcome domain, studies were judged to have low applicability concerns. Detailed PROBAST assessments are provided in Table 4.

**Table 4:** Risk of bias and applicability assessment of included prediction models, stratified by outcome type (SAD vs. SAE).

Study	ROB									Applicability			Overall	
	Participants	Predictors	Outcome	Analysis						Participants	Predictors	Outcome	ROB	Applicability
				EPV ≥ 20	Continuous variable	Missing data	Variable selection	Model performance	Model validation					
Gu Q (12)	−	?	?	+	+	−	+	+	+	+	+	+	−	+
Zhang Y (2)	−	?	?	−	+	+	+	+	+	+	+	+	−	+
Li (13)	−	?	?	−	+	−	+	+	+	+	+	+	−	+
Yang (14)	−	?	?	−	−	−	+	+	−	+	+	+	−	+
Jin J (15)	−	?	+	+	+	+	+	+	+	+	−	+	−	−
Zhao L (16)	−	?	?	+	+	−	+	+	+	+	+	+	−	+
Mei J (17)	+	+	+	−	+	−	+	+	+	+	+	+	−	+
Lu X (18)	−	?	?	−	+	+	−	−	+	+	−	+	−	−
Zhao Q (19)	−	?	?	+	+	−	+	+	+	+	+	+	−	+
Wang (20)	−	?	?	−	+	−	+	+	+	+	+	+	−	+
Zhou (21)	−	?	?	−	−	−	+	+	+	+	+	+	−	+
Zhang (22)	−	?	?	−	+	−	+	−	−	−	−	+	−	−

+ indicates low risk of bias/high applicability; − high risk of bias/low applicability? It is not clear. The overall risk of bias was judged as “high” if at least one domain was rated as high risk; it was judged as “low” only if all domains were rated as low risk; otherwise, the overall judgment was “unclear”.

## 4 Discussion

### 4.1 Current status and translational limitations of SABD risk prediction models

Existing SABD risk prediction models are mainly derived from ICU populations with sepsis, using retrospective cohort data to estimate the risk of developing impaired consciousness or delirium-related brain dysfunction. These studies are generally designed with the aim of early identification of potentially high-risk patients and represent exploratory work to assess the feasibility of such risk modeling. However, this systematic review found that, despite the increasing number of models, their findings remain limited in terms of stability, reproducibility, and clinical translatability. In view of the differences in outcome definitions, the methodological characteristics and limitations of SAD-based and SAE-based models are discussed separately below.

Risk prediction models that use SAD as the outcome typically rely on standardized delirium assessment tools such as CAM-ICU, with outcome assessment conducted within a predefined time window after ICU admission. As a result, the study population is restricted to a clinical syndrome that can be identified over a relatively short period (23, 24). Influenced by their retrospective design, these models are mainly built on routinely collected, structured clinical data available at or shortly after ICU admission, which inevitably places the models on a static or quasi-static snapshot of information and makes it difficult to capture key clinical contextual changes and temporal dynamics during the development of delirium (2, 12–14). At the same time, because only a small number of studies have been included, information on potential predictors is limited and currently remains largely at a methodological descriptive level. Age and SOFA score are repeatedly included in SAD models, which likely reflects their widespread availability and relatively consistent recording in routine ICU databases, rather than a proven stable or specific association with delirium. This modeling pathway means that, although delirium is a highly dynamic and context-dependent process, existing SAD prediction models capture these features only indirectly, which may limit their reproducibility and translatability across ICUs with different management practices and assessment protocols.

Risk prediction models that use SAE as the outcome generally adopt relatively broad operational definitions, most commonly based on reduced level of consciousness (e.g., GCS < 15 or ≤14) or documented abnormalities in mental or consciousness status in the medical record, so that the prediction target is oriented more toward identifying overall impairment of brain function (25). The development of existing SAE models likewise mainly relies on routine baseline clinical data that have already been recorded in existing clinical databases before outcome assessment. However, unlike SAD models, which depend on standardized assessment tools such as CAM-ICU, the outcome determination for SAE lacks unified operational criteria and is primarily based on crude grading of consciousness or unstructured narrative documentation in the medical record (15–22). The heterogeneity in case identification standards across studies limits the comparability of model results. At the level of candidate predictors, variables such as age, body temperature, SOFA score, APACHE II score, and serum sodium are repeatedly included in SAE models. This repetition should not be interpreted as evidence that these variables possess clear or stable predictive value; rather, it more likely reflects common patterns of variable selection shaped by data availability and recording structures under specific methodological constraints in previous studies. Consequently, the incorporation of specialized assessments or monitoring indicators that more directly reflect central nervous system status remains limited in current models, resulting in insufficient direct characterization of brain dysfunction itself.

Although SAD and SAE models differ in their outcome definitions, the two types of models share similar structural features in their modeling pathways: both rely on retrospective clinical databases and use routinely recorded basic clinical data as the primary source of modeling information. Variable selection is constrained by data availability rather than being theoretically driven by mechanisms of brain dysfunction, which fundamentally limits the models’ ability to characterize central nervous system involvement. In addition, before clearly specifying the exact outcome type targeted by a model and aligning it with existing ICU consciousness assessment procedures and delirium screening protocols, it is difficult to integrate model outputs into clinical workflows. These structural methodological issues help explain why current SABD risk prediction models remain difficult to replicate or translate into practice. Given the fundamental differences between SAD and SAE in measurement tools, assessment time windows, and misclassification structures, current evidence is also insufficient to support the development of a single unified prediction framework under the umbrella concept of SABD. Therefore, future studies should, on the basis of clearly defined outcome constructs, establish relatively standardized processes for outcome definition, predictor selection, data handling, and model validation separately for SAD and SAE, progressively incorporate assessment or monitoring information that more directly reflects central nervous system status, and consider their alignment with clinical assessment workflows already at the stage of model development.

### 4.2 High risk of bias in SABD risk prediction models

This systematic review included 12 studies, of which 4 used SAD as the outcome and 8 used SAE. Although the two types of models differed in their outcome definitions, the PROBAST assessment showed that the sources and distribution of risk of bias were similar, and all included studies were at high risk of bias. Therefore, they were analyzed jointly. In the participants domain, 11 studies collected and analyzed data retrospectively, which may have affected data completeness and accuracy, thereby resulting in a high risk of bias (2, 12–16, 18–22). Future studies should prioritize prospective cohort designs, nested case–control studies, or case-cohort studies to reduce the risk of data-related bias. In the predictors domain, 11 studies did not clearly report whether the measurement of predictors was consistent with the outcome definition, nor whether blinding was applied during predictor assessment, leading to an unclear risk of bias rating in this domain (2, 12–16, 18–22). Future research should explicitly clarify the consistency between predictor measurement and outcome definition and strictly adhere to blinding procedures to minimize assessment bias. In the outcome domain, 10 studies failed to report whether outcome definitions were prespecified, resulting in an unclear risk of bias assessment (2, 12–14, 16, 18–22). Future studies should prespecify outcome definitions and clearly describe them in study protocols to ensure standardized and reproducible outcome assessment.

In the analysis domain: (1) Inadequate number of outcome events. The adequacy of outcome events is commonly assessed using EPV. PROBAST recommends (10) that during model development, the ratio of outcome events to the number of candidate predictors should be EPV ≥ 20, and during model validation, the number of outcome events should be≥100, in order to minimize the risk of overfitting. In this review, four studies met these criteria (12, 15, 16, 19), whereas eight studies reported EPV < 20 (2, 13, 14, 17, 18, 20–22), indicating a potential risk of overfitting. Future studies are therefore encouraged to optimize sample size planning (EPV ≥ 20) or to adopt the machine learning-oriented sample size calculation approach proposed by Riley (26), thereby reducing the risk of bias. (2) Variable selection. Lu X (18) selected predictors based on univariable analyses, which may overlook interactions and underlying relationships among multiple variables, potentially leading to omission of important predictors and introducing bias. Previous studies have shown that penalized regression methods such as LASSO regression, Ridge regression, and Elastic Net regression can help reduce the risk of overfitting (27). Future research should incorporate clinical expertise and apply these approaches appropriately for variable selection to improve the rigor and standardization of the model development process. (3) Inappropriate handling of continuous variables. Yang (14) and Zhou (21) categorized age, which may result in substantial information loss from continuous variables and consequently introduce bias. Future studies are recommended to retain numerical variables in their original continuous form or, if categorization is necessary, to convert them into categorical variables with more than four levels, rather than dichotomous variables, in order to reduce information loss and model distortion (10). (4) Handling of missing data. Eight studies did not report how missing data were handled (12–14, 16, 17, 19–22), and Zhao Q (19) directly excluded observations with missing values. Such complete case exclusion of otherwise eligible participants may introduce bias in the associations between predictors and outcomes, thereby distorting model performance. PROBAST recommends multiple imputation as the most appropriate approach for handling missing data, as it can effectively mitigate the adverse effects of missingness on statistical analysis and model stability and improve the reliability of model results (28). Future studies are therefore recommended to report the proportion and mechanism of missing data for each variable and to use multiple imputation whenever possible for data handling. (5) Incomplete evaluation of model calibration. The predictive performance of a model is typically comprehensively assessed by evaluating both model calibration and discrimination, which together determine the applicability of predicted risks. Lu X (18) and Zhang (22) did not report model calibration, making it difficult to determine the model’s ability to accurately estimate individual outcome probabilities; therefore, these studies were judged to be at high risk of bias. Future studies are recommended to strictly adhere to established reporting standards for risk prediction models, in order to reduce the risk of model bias and improve transparency and reliability. (6) Lack of model validation. Internal validation assesses the stability of a model within the development dataset and detects the risk of overfitting, whereas external validation evaluates the applicability and generalizability of the model in independent external datasets. Two studies (14, 22) reported model development without any validation. In such cases, model performance metrics are more susceptible to random error, and both model stability and generalizability remain uncertain. Future studies are recommended to conduct at least internal validation after model development, preferably using resampling methods such as bootstrap or cross-validation. External validation should then be performed in independent cohorts, and model recalibration should be undertaken when necessary before considering broader application.

In summary, the high risk of bias was mainly concentrated in inadequate number of outcome events, variable selection, inappropriate handling of continuous variables, handling of missing data, incomplete evaluation of model calibration, and lack of model validation. Future studies should therefore strictly adhere to the PROBAST framework and the Transparent Reporting of a Multivariable Prediction Model for Individual Prognosis or Diagnosis (TRIPOD) statement (29) in both model development and reporting, in order to better control the risk of bias, standardize reporting practices, and ultimately enhance the reliability and clinical translatability of SABD risk prediction models.

### 4.3 Limitations

The limitations of this study include the following: (1) Limited population representativeness: the included studies were mainly from a small number of countries and institutions, mostly single-center studies or based on specific databases, making it difficult to fully reflect prediction modeling practices across different regions, hospital levels, and clinical pathways. (2) Insufficient information on external validation: most studies only conducted internal validation or did not report validation, making it difficult to further examine the impact of different validation strategies on model robustness and reproducibility. (3) Limited overall methodological quality of the included studies: there were common shortcomings in study design, variable selection, data handling, and modeling methods, all included studies were assessed as having a high risk of bias. Therefore, we did not perform a quantitative synthesis of the results but instead summarized and discussed them primarily from a qualitative methodological perspective.

## 5 Conclusion

This study systematically reviewed 24 SABD risk prediction models from 12 studies from a methodological perspective. The findings suggest that current prediction model research remains at an exploratory stage. Most models reported discrimination as the primary performance metric. However, in the context of high risk of bias and insufficient validation, these results only reflect apparent model performance and do not provide reliable evidence of true predictive ability or clinical applicability. Future studies on risk prediction models should strictly adhere to PROBAST and TRIPOD, conduct large multicenter prospective studies, and establish standardized validation procedures to improve methodological quality and potential translational value.

## Data Availability

The original contributions presented in the study are included in the article/Supplementary material, further inquiries can be directed to the corresponding authors.
