# Supplementary material for: Systematic review of risk prediction models for sepsis-associated brain dysfunction
Source: Front Neurol. 2026 Feb 27;17:1653460. doi: 10.3389/fneur.2026.1653460 (PMC12982069; doi:10.3389/fneur.2026.1653460)
Supplement: Supplementary file 1 [file Data_Sheet_1.zip › Supplementary-TRIPOD/TRIPOD adherence table.pdf]

TRIPOD adherence table

Study	Title and abstract				Methods																
	Title	Abstract	Background		Source of data		Participants			Outcome		Predictors		Sample size	Missing data	Statistical analysis methods					Risk groups	D vs.v
	1	2	3a	3b	4a	4b	5a	5b	5c	6a	6b	7a	7b	8	9	10a	10b	10c	10d	10e	11	12
Gu Q[12]	N	N	Y	Y	Y	N	Y	N	N	N	N	N	N	Y	N	N	Y	NA	Y	NA	N	NA
Zhang Y[2]	Y	N	Y	Y	Y	N	Y	Y	N	Y	N	N	N	Y	Y	N	Y	N	Y	N	N	N
Jin J[13]	N	N	Y	Y	Y	N	Y	Y	N	Y	N	N	N	N	Y	N	Y	NA	Y	NA	N	NA
Ge C[14]	N	N	Y	Y	Y	N	Y	Y	Y	N	N	N	N	Y	Y	N	Y	NA	Y	NA	N	NA
Zhao L[15]	N	N	Y	Y	Y	N	Y	Y	N	Y	N	N	N	Y	N	N	Y	NA	Y	NA	N	NA
Mei J[16]	Y	N	Y	Y	Y	Y	Y	Y	N	N	N	Y	N	Y	N	N	Y	NA	Y	NA	N	NA
Lu X[17]	N	N	Y	Y	Y	N	Y	Y	N	N	N	N	N	Y	Y	N	Y	NA	N	NA	N	NA
Zhao Q[18]	Y	Y	N	Y	Y	N	Y	Y	N	N	N	N	N	N	N	N	Y	NA	Y	NA	N	NA
Gu[19]	Y	N	N	Y	Y	Y	Y	Y	N	N	N	N	N	N	N	N	N	NA	Y	NA	N	NA
Li[20]	N	N	Y	Y	Y	Y	Y	Y	N	N	N	N	N	N	N	N	Y	NA	Y	NA	N	NA
Wang[21]	Y	Y	N	Y	Y	Y	Y	Y	N	N	N	N	N	N	N	N	N	NA	Y	NA	N	NA
Yang[22]	N	Y	N	Y	Y	N	Y	Y	N	N	N	N	N	N	N	N	N	NA	Y	NA	N	NA
Huang[23]	N	N	N	N	Y	N	Y	Y	N	N	N	N	N	Y	N	N	Y	NA	Y	NA	N	NA
Zhou[24]	Y	Y	N	Y	Y	Y	Y	Y	N	N	N	N	N	N	N	N	Y	NA	Y	NA	N	NA
Zhang[25]	Y	N	Y	Y	Y	N	Y	Y	N	N	N	N	N	N	N	N	N	NA	N	NA	N	NA

Note:yes (Y), no (N), referenced (R), and not applicable (NA);Firstly, the compliance of the report was calculated item by item according to the TRIPOD project. If all the elements of the compliance of a TRIPOD project were answered as "Yes", the compliance score of that project would be "1", but if any one of them was "No", it would be "0". In some cases, different scoring rules were used, and the specific rules were explained for the corresponding projects in the compliance assessment table below.

Continued TRIPOD adherence table

**Study**	Results									Discussion				Other information		**Score**
	**Participants**			**Model development**		**Model specification**		**Model performance**	**Model updating**	**Limitations**	**Interpretation**		**Implications**	**Supplementary information**	**Funding**	
	13a	13b	13c	14a	14b	15a	15b	16	17	18	19a	19b	20	21	22	
Gu Q[12]	N	N	NA	Y	Y	N	Y	Y	NA	Y	NA	Y	Y	Y	Y	53.33%
Zhang Y[2]	N	N	Y	N	Y	Y	Y	N	N	Y	Y	Y	Y	Y	Y	55.56%
Jin J[13]	N	N	NA	N	N	N	Y	Y	NA	Y	NA	Y	Y	N	Y	50%
Ge C[14]	NA	N	NA	N	Y	N	Y	N	NA	Y	NA	Y	Y	Y	Y	58.62%
Zhao L[15]	N	N	NA	N	Y	N	Y	Y	NA	Y	NA	Y	Y	Y	Y	53.33%
Mei J[16]	N	N	NA	N	Y	Y	Y	Y	NA	Y	NA	Y	Y	N	Y	63.33%
Lu X[17]	N	N	NA	N	Y	N	Y	N	NA	Y	NA	Y	Y	Y	Y	46.67%
Zhao Q[18]	N	N	NA	Y	Y	N	Y	Y	NA	Y	NA	Y	Y	Y	Y	53.33%
Gu[19]	N	N	NA	Y	N	N	Y	N	NA	Y	NA	Y	Y	N	Y	43.33%
Li[20]	N	N	NA	Y	Y	N	Y	Y	NA	Y	NA	Y	Y	N	Y	53.33%
Wang[21]	N	N	NA	Y	N	N	Y	Y	NA	Y	NA	Y	Y	N	Y	50%
Yang[22]	N	N	NA	Y	Y	N	Y	Y	NA	Y	NA	Y	Y	N	N	43.33%
Huang[23]	N	N	NA	Y	Y	N	Y	Y	NA	Y	NA	Y	Y	N	Y	46.67%
Zhou[24]	N	N	NA	Y	Y	N	Y	Y	NA	Y	NA	Y	Y	N	Y	56.67%
Zhang[25]	N	N	NA	Y	Y	N	N	N	NA	Y	NA	Y	Y	N	Y	40%

Note:yes (Y), no (N), referenced (R), and not applicable (NA);Firstly, the compliance of the report was calculated item by item according to the TRIPOD project. If all the elements of the compliance of a TRIPOD project were answered as "Yes", the compliance score of that project would be "1", but if any one of them was "No", it would be "0". In some cases, different scoring rules were used, and the specific rules were explained for the corresponding projects in the compliance assessment table below.

1	Identify the study as developing and/or validating a multivariable prediction model, the target population, and the outcome to be predicted.
2	Provide a summary of objectives, study design, setting, participants, sample size, predictors, outcome, statistical analysis, results, and conclusions.
3a	Explain the medical context (including whether diagnostic or prognostic) and rationale for developing or validating the multivariable prediction model, including references to existing models.
3b	Specify the objectives, including whether the study describes the development or validation of the model or both.
4a	Describe the study design or source of data (e.g., randomized trial, cohort, or registry data), separately for the development and validation data sets, if applicable.
4b	Specify the key study dates, including start of accrual; end of accrual; and, if applicable, end of follow-up.
5a	Specify key elements of the study setting (e.g., primary care, secondary care, general population) including number and location of centres.
5b	Describe eligibility criteria for participants.
5c	Give details of treatments received, if relevant.
6a	Clearly define the outcome that is predicted by the prediction model, including how and when assessed.
6b	Report any actions to blind assessment of the outcome to be predicted.
7a	Clearly define all predictors used in developing or validating the multivariable prediction model, including how and when they were measured.
7b	Report any actions to blind assessment of predictors for the outcome and other predictors.
8	Explain how the study size was arrived at.
9	Describe how missing data were handled (e.g., complete-case analysis, single imputation, multiple imputation) with details of any imputation method.
10a	Describe how predictors were handled in the analyses.
10b	Specify type of model, all model-building procedures (including any predictor selection), and method for internal validation.
10c	For validation, describe how the predictions were calculated.
10d	Specify all measures used to assess model performance and, if relevant, to compare multiple models.[2] *These should be described in the methods section of the paper (item 16 addresses the reporting of the results for model performance).*
10e	Describe any model updating (e.g., recalibration) arising from the validation, if done.

11	Provide details on how risk groups were created, if done.
12	For validation, identify any differences from the development data in setting, eligibility criteria, outcome and predictors.
13a	Describe the flow of participants through the study, including the number of participants with and without the outcome and, if applicable, a summary of the follow-up time. A diagram may be helpful.
13b	Describe the characteristics of the participants (basic demographics, clinical features, available predictors), including the number of participants with missing data for predictors and outcome.
13c	For validation, show a comparison with the development data of the distribution of important variables (demographics, predictors and outcome).
14a	Specify the number of participants and outcome events in each analysis.
14b	If done, report the unadjusted association between each candidate predictor and outcome.
15a	Present the full prediction model to allow predictions for individuals (i.e., all regression coefficients, and model intercept or baseline survival at a given time point).
15b	Explain how to use the prediction model.
16	Report performance measures (with confidence intervals) for the prediction model.[3]<br>*These should be described in results section of the paper (item 10 addresses the reporting of the methods for model performance).*
17	If done, report the results from any model updating (i.e., model specification, model performance, recalibration). *If updating was not done, score this TRIPOD item as、Not applicable,.*
18	Discuss any limitations of the study (such as nonrepresentative sample, few events per predictor, missing data).
19a	For validation, discuss the results with reference to performance in the development data, and any other validation data.
19b	Give an overall interpretation of the results considering objectives, limitations, results from similar studies and other relevant evidence.
20	Discuss the potential clinical use of the model and implications for future research.
21	Provide information about the availability of supplementary resources, such as study protocol, web calculator, and data sets.
22	Give the source of funding and the role of the funders for the present study.

Note:Items not included in the total score are highlighted in yellow.
