# Supplementary material for: Systematic review of risk prediction models for sepsis-associated brain dysfunction
Source: Front Neurol. 2026 Feb 27;17:1653460. doi: 10.3389/fneur.2026.1653460 (PMC12982069; doi:10.3389/fneur.2026.1653460)
Supplement: Supplementary file 1 [file Data_Sheet_1.zip › Supplementary-TRIPOD/TRIPOD-16 Mei J 2024.docx]

**Assessing adherence of prediction model reports to the TRIPOD guideline**

**This document provides guidance for extracting the relevant information and calculating summary scores to determine adherence of primary prediction**

**model reports to the TRIPOD (Transparent Reporting of studies on prediction models for Individual Prognosis Or Diagnosis) reporting guideline (issued in**

**January 2015; [www.tripod-statement.org](https://www.tripod-statement.org)). To be able to compare TRIPOD adherence evaluations, e.g. over time or over clinical domains, it is crucial that investigators use uniform methods, i.e. this adherence assessment form. If investigators decide to deviate from this form and scoring rules, they should be explicit and transparent about the changes they make.**

**Extracting the data**

**This TRIPOD adherence assessment form consists of two parts. Part A is to extract general information from a publication about the development and/or**

**validation of a diagnostic or prognostic prediction model, or about the assessment of the incremental value of one or more predictors on top of an existing prediction model. Part B lists all 22 main items of the original TRIPOD reporting guideline, of which ten were divided in sub items (denoted by a, b, c, etc.). Below, these are shaded in blue and further referred to as the TRIPOD items. To properly assess adherence of a study report to the TRIPOD reporting items, we further specified these TRIPOD items into multiple so-called adherence elements (denoted by i, ii, iii, …) simply because the original TRIPOD items often mentioned multiple elements to report. Accordingly, the form below provides a comprehensive tool to look for the information deemed necessary by the TRIPOD reporting guideline to judge the adherence of reports to this guideline.**

**There are four columns in which information can be entered: one for reports about the development of a prediction model [D], one for reports on external validation of a prediction model [V], one for reports on the incremental value of predictor(s) to an existing prediction model [IV], and one for reports on the development plus external validation of the same model [D+V]. If a report addresses both the development and validation of the same model, then both**

**columns D and V should be used to assess the reporting of the development and external validation, and, subsequently, column D+V to combine the**

**information of these two. If a report addresses the development of a model and external validation of a different model, one can use the columns D and V to assess the reporting however, information should not be combined using column D+V. For publications in which more than one (different) prediction model is developed or validated, scoring could be based on the model of interest (or most clearly reported model).**

**The adherence elements are formulated as statements, for which there are four potential answer options: yes (Y), no (N), referenced (R), and not applicable (NA). For some elements it may be acceptable if authors in their report specifically reference to another publication (i.e. explicitly mention that the**

**information of that adherence element is described somewhere else). This is denoted by the answer option “R” . For adherence elements that do not apply to a specific situation, there is the answer option “NA” .**

**Some TRIPOD items do not apply to all four types of prediction model studes, e.g. TRIPOD item 10a “Describe how predictors were handled in the analyses”, is not applicable when reporting about external validation, whereas TRIPOD item 10c “For validation, describe how the predictions were calculated” does not apply to the reporting of model development. In such instances we state ‘not applicable’ and grey shaded these adherence elements.**

**Calculating adherence to TRIPOD**

**First, adherence of a report is calculated per TRIPOD item. If the answer to all adherence elements of a particular TRIPOD item is scored “yes”, adherence to that TRIPOD item is scored as “1”, and non adherence as “0” . In some situations a different scoring rule is used, which is described in the adherence**

**assessment form below for the corresponding items.**

**Subsequently, a report’s overall TRIPOD adherence score can be calculated. This is calculated by dividing the sum of the adhered TRIPOD items by the total number of applicable TRIPOD items for that report. This total can vary since some TRIPOD items may be not applicable to all four types of prediction model studies. The total number of applicable TRIPOD items for D studies is 30, for V 30, for D+V 36 and for IV 35.1 In addition, five TRIPOD items (5c, 10e, 11, 14b, and 17) might not be applicable for specific reports.**

**If one reviews multiple prediction model studies on their adherence to TRIPOD, overall adherence per TRIPOD item can be calculated by dividing the number of studies that adhered to a specific TRIPOD item by the number of studies in which the specific TRIPOD item was applicable.**


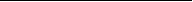


**1 TRIPOD item 21 is not taken into account in the overall score in any of the four types of studies.**

| **A. GENERAL INFORMATION** | |
| --- | --- |
| **Study ID** |  |
| **First author** |  |
| **Publication year** |  |
| **Title** | Mei J, Zhang X, Sun X, Hu L, Song Y. Optimizing the prediction of sepsis-associated encephalopathy with cerebral circulation time utilizing a nomogram: a pilot study in the intensive care unit. Front Neurol. 2024 Jan 11;14:1303075. DOI: 10.3389/fneur.2023.1303075. |
| **Journal** |  |
| **Diagnostic or**  **prognostic**  **prediction model?** | **□ Diagnostic**  **□ Prognostic** |
| **Type of**  **prediction model study**  ***(multiple options possible)*** | **□ Development**  **□ External validation**  ***If both development and external validation:***  **□ same model/score**  **□ different models/scores**  **□ Incremental value** |

| **B. TRIPOD ITEMS** | | | | | | |
| --- | --- | --- | --- | --- | --- | --- |
|  | | | **[D]**  **Development** | **[V]**  **External**  **validation** | **[IV]**  **Incremental**  **value** | **[D+V]**  **Development**  **and external**  **validation (of**  **same model)** |
| **Title and abstract**  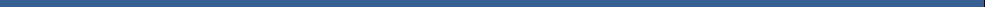  ***It is suggested to score items 1 and 2 (Title and Abstract) after scoring items 3 to 22, as only after reading the whole publication it can be judged whether the reporting in the title and abstract is complete.*** | | | | | | |
| **Title** | **1** | **Identify the study as developing and/or validating a multivariable prediction model, the target population, and the outcome to be**  **predicted.** | **Score 1 if all**  **elements are**  **scored as “Y”** | **Score 1 if all**  **elements are**  **scored as “Y”** | **Score 1 if all**  **elements are**  **scored as “Y”** | **Score 1 if all**  **elements are**  **scored as “Y”** |
|  | **i** | **The words developing/development, validation/validating, incremental/added value (or synonyms) are reported in the title** | **Y** | **Y / N** | **Y / N** | **=Y if D1i=Y AND V1i=Y** |
|  | **ii** | **The words prediction, risk prediction, prediction model, risk models, prognostic models, prognostic indices, risk scores (or synonyms) are reported in the title** | **Y** | **Y / N** | **Y / N** | **=Y if D1ii=Y OR V1ii=Y** |
|  | **iii** | **The target population is reported in the title** | **Y** | **Y / N** | **Y / N** | **=Y if D1iii=Y OR V1iii=Y** |
|  | **iv** | **The outcome to be predicted is reported in the title** | **Y** | **Y / N** | **Y / N** | **=Y if D1iv=Y OR V1iv=Y** |
| **Abstract** | **2** | **Provide a summary of objectives, study design, setting, participants, sample size, predictors, outcome, statistical analysis, results, and**  **conclusions.** | **Score 1 if all**  **elements are**  **scored as “Y” or “NA”** | **Score 1 if all**  **elements are**  **scored as “Y” or “NA”** | **Score 1 if all**  **elements are**  **scored as “Y” or “NA”** | **Score 1 if all**  **elements are**  **scored as “Y” or “NA”** |
|  | **i** | **The objectives are reported in the abstract** | **Y** | **Y / N** | **Y / N** | **=Y if D2i=Y AND V2i=Y** |
|  | **ii** | **Sources of data are reported in the abstract**  ***E.g. Prospective cohort, registry data, RCT data.*** | **Y** | **Y / N** | **Y / N** | **=Y if D2ii=Y AND V2ii=Y** |
|  | **iii** | **The setting is reported in the abstract**  ***E.g. Primary care, secondary care, general population, adult care, or paediatric care. The setting should be reported for both the development and validation datasets, if applicable.*** | **Y** | **Y / N** | **Y / N** | **=Y if D2iii=Y AND V2iii=Y** |
|  | **iv** | **A general definition of the study participants is reported in the abstract**  ***E.g. patients with suspicion of certain disease, patients with a specific disease, or general eligibility criteria.*** | **Y** | **Y / N** | **Y / N** | **=Y if D2iv=Y AND V2iv=Y** |
|  | **v** | **The overall sample size is reported in the abstract** | **Y** | **Y / N** | 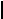**Y / N** | **=Y if D2v=Y AND** |

|  |  |  |  |  |  | **V2v=Y** |
| --- | --- | --- | --- | --- | --- | --- |
|  | **vi** | **The number of events (or % outcome together with overall sample size) is reported in the abstract**  ***If*** ***a*** ***continuous*** ***outcome*** ***was*** ***studied,*** ***score*** ***Not*** ***applicable*** | **Y** | **Y / N / NA** | **Y / N / NA** | **=Y if (D2vi=Y AND V2vi=(Y OR NA)) OR (D2vi = (Y OR NA)**  **AND V2vi=Y)**  **=NA if D2vi=NA AND V2vi=NA** |
|  | **vii** | **Predictors included in the final model are reported in the abstract. For validation studies of well-known models, at least the name/acronym of the validated**  **model is reported**  ***Broad*** ***descriptions*** ***are*** ***sufficient,*** ***e.g.*** ***、all*** ***information*** ***from*** ***patient*** ***history*** ***and*** ***physical*** ***examination,.***  ***Check*** ***in*** ***the*** ***main*** ***text*** ***whether*** ***all*** ***predictors*** ***of*** ***the*** ***final*** ***model*** ***are*** ***indeed*** ***reported*** ***in*** ***the*** ***abstract.*** | **N** | **Y / N** | **Y / N** | **=Y if D2vii=Y OR V2vii=Y** |
|  | **viii** | **The outcome is reported in the abstract** | **Y** | **Y / N** | **Y / N** | **=Y if D2viii=Y AND V2viii=Y** |
|  | **ix** | **Statistical methods are described in the abstract**  ***For*** ***model*** ***development,*** ***at*** ***least*** ***the*** ***type*** ***of*** ***statistical*** ***model*** ***should*** ***be***  ***reported.*** ***For*** ***validation*** ***studies*** ***a*** ***quote*** ***like“model,s*** ***discrimination*** ***and***  ***calibration*** ***was*** ***assessed”is*** ***considered*** ***adequate.*** ***If*** ***done,*** ***methods*** ***of*** ***updating*** ***should*** ***be*** ***reported.*** | **N** | **Y / N** | **Y / N** | **=Y if D2ix=Y AND V2ix=Y** |
|  | **x** | **Results for model discrimination are reported in the abstract**  ***This*** ***should*** ***be*** ***reported*** ***separately*** ***for*** ***development*** ***and*** ***validation*** ***if*** ***a*** ***study*** ***includes*** ***both*** ***development*** ***and*** ***validation.*** | **Y** | **Y / N** | **Y / N** | **=Y if D2x=Y AND V2x=Y** |
|  | **xi** | **Results for model calibration are reported in the abstract**  ***This*** ***should*** ***be*** ***reported*** ***separately*** ***for*** ***development*** ***and*** ***validation*** ***if*** ***a*** ***study*** ***includes*** ***both*** ***development*** ***and*** ***validation.*** | **N** | **Y / N** | **Y / N** | **=Y if D2xi=Y AND V2xi=Y** |
|  | **xii** | **Conclusions are reported in the abstract**  ***In*** ***publications*** ***addressing*** ***both*** ***model*** ***development*** ***and*** ***validation,*** ***there*** ***is*** ***no*** ***need*** ***for*** ***separate*** ***conclusions*** ***for*** ***both;*** ***one*** ***conclusion*** ***is*** ***sufficient.*** | **Y** | **Y / N** | **Y / N** | **=Y if D2xii=Y OR V2xii=Y** |
| **Background**  **and objectives** | **3a** | **Explain the medical context (including whether diagnostic or**  **prognostic) and rationale for developing or validating the**  **multivariable prediction model, including references to existing models.** | **Score 1 if both**  **elements are**  **scored as “Y”** | **Score 1 if both**  **elements are**  **scored as “Y”** | **Score 1 if both**  **elements are**  **scored as “Y”** | **Score 1 if both**  **elements are**  **scored as “Y”** |
|  | **i** | **The background and rationale are presented** | **Y** | **Y / N** | **Y / N** | **=Y if D3ai=Y OR V3ai=Y** |
|  | **ii** | **Reference to existing models is included (or stated that there are no existing models)** | **Y** | **Y / N** | **Y / N** | **=Y if D3aii=Y OR V3aii=Y** |
|  | **3b** | **Specify the objectives, including whether the study describes the development or validation of the model or both.** | **Score 1 if**  **element is**  **scored as “Y”** | **Score 1 if**  **element is**  **scored as “Y”** | **Score 1 if**  **element is**  **scored as “Y”** | **Score 1 if element is scored as “Y”** |

|  | **i** | **It is stated whether the study describes development and/or validation and/or incremental (added) value** | **Y** | **Y / N** | **Y / N** | **=Y if D3bi=Y AND V3bi=Y** |
| --- | --- | --- | --- | --- | --- | --- |
| **Methods** |  |  |  |  |  |  |
| **Source of data** | **4a** | **Describe the study design or source of data (e.g., randomized trial, cohort, or registry data), separately for the development and**  **validation data sets, if applicable.** | **Score 1 if**  **element is**  **scored as “Y”** | **Score 1 if**  **element is**  **scored as “Y”** | **Score 1 if**  **element is**  **scored as “Y”** | **Score 1 if element is scored as “Y”** |
|  | **i** | **The study design/source of data is described**  ***E.g. Prospectively designed, existing cohort, existing RCT, registry/medical records, case control, case series.***  ***This needs to be explicitly reported; reference to this information in another article alone is insufficient.*** | **Y** | **Y / N** | **Y / N** | **=Y if D4ai=Y AND V4ai=Y** |
|  | **4b** | **Specify the key study dates, including start of accrual; end of accrual; and, if applicable, end of follow-up.** | **Score 1 if all**  **elements are**  **scored as ”Y”, “NA”, or “R”** | **Score 1 if all**  **elements are**  **scored as ”Y”, “NA”, or “R”** | **Score 1 if all**  **elements are**  **scored as ”Y”, “NA”, or “R”** | **Score 1 if all**  **elements are**  **scored as ”Y”, “NA”, or “R”** |
|  | **i** | **The starting date of accrual is reported** | **Y** | **Y / N / R** | **Y / N / R** | **=Y if (D4bi=Y AND V4bi=(Y OR R)) OR (D4bi =(Y OR R)**  **AND V4bi=Y)**  **=R if D4bi=R AND V4bi=R** |
|  | **ii** | **The end date of accrual is reported** | **Y** | **Y / N / R** | **Y / N / R** | **=Y if (D4bii=Y AND V4bii=(Y OR R)) OR (D4bii =(Y OR R)**  **AND V4bii=Y)**  **=R if D4bii=R AND V4bii=R** |
|  | **iii** | **The length of follow-up and prediction horizon/time frame are reported, if applicable**  ***E.g. “Patients were followed from baseline for 10 years“ and “10-year prediction of…”; notably for prognostic studies with long term follow-up.***  ***If this is not applicable for an article (i.e. diagnostic study or no follow-up), then score Not applicable.*** | **Y** | **Y / N / NA** | **Y / N / NA** | **=Y if (D4biii=Y AND V4biii=(Y OR NA)) OR (D4biii =(Y OR NA) AND V4biii=Y)**  **=NA if D4biii=NA AND V4biii=NA** |
| **Participants** | **5a** | 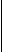**Specify key elements of the study setting (e.g., primary care,**  **secondary care, general population) including number and location of centres.** | **Score 1 if all**  **elements are**  **scored as “Y” or “R”** | **Score 1 if all**  **elements are**  **scored as “Y” or “R”** | **Score 1 if all**  **elements are**  **scored as “Y” or “R”** | **Score 1 if all**  **elements are**  **scored as “Y” or “R”** |

|  | **i** | **The study setting is reported (e.g. primary care, secondary care, general population)**  ***E.g. : ‘surgery for endometrial cancer patients’ is considered to be enough information about the study setting.*** | **Y** | **Y / N / R** | **Y / N / R** | **=Y if (D5ai=Y AND V5ai=(Y OR R)) OR (D5ai =(Y OR R)**  **AND V5ai=Y)**  **=R if D5ai=R AND V5ai=R** |
| --- | --- | --- | --- | --- | --- | --- |
|  | **ii** | **The number of centres involved is reported**  ***If the number is not reported explicitly, but can be concluded from the name of the centre/centres, or if clearly a single centre study, score Yes.*** | **Y** | **Y / N / R** | **Y / N / R** | **=Y if (D5aii=Y AND V5aii=(Y OR R)) OR (D5aii =(Y OR R)**  **AND V5aii=Y)**  **=R if D5aii=R AND V5aii=R** |
|  | **iii** | **The geographical location (at least country) of centres involved is reported**  ***If no geographical location is specified, but the location can be concluded from the name of the centre(s), score Yes.*** | **Y** | **Y / N / R** | **Y / N / R** | **=Y if (D5aiii=Y AND V5aiii=(Y OR R)) OR (D5aiii =(Y OR R)**  **AND V5aiii=Y)**  **=R if D5aiii=R AND V5aiii=R** |
|  | **5b** | **Describe eligibility criteria for participants.** | **Score 1 if**  **element is**  **scored as “Y”** | **Score 1 if**  **element is**  **scored as “Y”** | **Score 1 if**  **element is**  **scored as “Y”** | **Score 1 if element is scored as “Y”** |
|  | **i** | **In-/exclusion criteria are stated**  ***These should explicitly be stated. Reasons for exclusion only described in a patient flow is not sufficient.*** | **Y** | **Y / N** | **Y / N** | **=Y if D5bi=Y AND V5bi=Y** |
|  | **5c** | **Give details of treatments received, if relevant.** | **Score 1 if**  **element is**  **scored as “Y”;**  **score *Not***  ***applicable* if**  **element is**  **scored as “NA”** | **Score 1 if**  **element is**  **scored as “Y”;**  **score *Not***  ***applicable* if**  **element is**  **scored as “NA”** | **Score 1 if**  **element is**  **scored as “Y”;**  **score *Not***  ***applicable* if**  **element is**  **scored as “NA”** | **Score 1 if element is scored as “Y”; score *Not***  ***applicable* if**  **element is scored as “NA”** |
|  | **i** | **Details of any treatments received are described**  ***This item is notably for prognostic modelling studies and is about treatment at baseline or during follow-up. The ‘if relevant’ judgment of treatment requires clinical knowledge and interpretation.***  ***If you are certain that treatment was not relevant, e.g. in some diagnostic model studies, score Not applicable*** | **N** | **Y / N / NA** | **Y / N / NA** | **=Y if (D5ci=Y AND V5ci=(Y OR NA) OR (D5ci=(Y OR NA)**  **AND V5ci=Y)**  **=NA if D5ci=NA AND V5ci=NA** |
| **Outcome** | **6a** | **Clearly define the outcome that is predicted by the prediction model, including how and when assessed.** | **Score 1 if all**  **elements are**  **scored as “Y” or “R”** | **Score 1 if all**  **elements are**  **scored as “Y” or “R”** | **Score 1 if all**  **elements are**  **scored as “Y” or “R”** | **Score 1 if all**  **elements are**  **scored as “Y” or “R”** |

|  | **i** | **The outcome definition is clearly presented**  ***This*** ***should*** ***be*** ***reported*** ***separately*** ***for*** ***development*** ***and*** ***validation*** ***if*** ***a*** ***publication*** ***includes*** ***both.*** | **Y** | **Y / N / R** | **Y / N / R** | **=Y if (D6ai=Y AND V6ai=(Y OR R)) OR (D6ai =(Y OR R)**  **AND V6ai=Y)**  **=R if D6ai=R AND V6ai=R** |
| --- | --- | --- | --- | --- | --- | --- |
|  | **ii** | **It is described how outcome was assessed (including all elements of any composite, for example CVD [e.g. MI, HF, stroke]) *.*** | **N** | **Y / N / R** | **Y / N / R** | **=Y if (D6aii=Y AND V6aii=(Y OR R)) OR (D6aii =(Y OR R)**  **AND V6aii=Y)**  **=R if D6aii=R AND V6aii=R** |
|  | **iii** | **It is described when the outcome was assessed (time point(s) since T0)** | **N** | **Y / N / R** | **Y / N / R** | **=Y if (D6aiii=Y AND V6aiii=(Y OR R)) OR (D6aiii =(Y OR R)**  **AND V6aiii=Y)**  **=R if D6aiii=R AND V6aiii=R** |
|  | **6b** | **Report any actions to blind assessment of the outcome to be predicted.** | **Score 1 if**  **element is**  **scored as “Y”** | **Score 1 if**  **element is**  **scored as “Y”** | **Score 1 if**  **element is**  **scored as “Y”** | **Score 1 if element is scored as “Y”** |
|  | **i** | **Actions to blind assessment of outcome to be predicted are reported**  ***If*** ***it*** ***is*** ***clearly*** ***a*** ***non-issue*** ***(e.g.*** ***all-cause*** ***mortality*** ***or*** ***an*** ***outcome*** ***not*** ***requiring*** ***interpretation),*** ***score*** ***Yes.*** ***In*** ***all*** ***other*** ***instances,*** ***an*** ***explicit*** ***mention*** ***is***  ***expected.*** | **N** | **Y / N** | **Y / N** | **=Y if D6bi=Y AND V6bi=Y** |
| **Predictors** | **7a** | **Clearly define all predictors used in developing or validating the**  **multivariable prediction model, including how and when they were measured.** | **Score 1 if all**  **elements are**  **scored as “Y” or “R”** | **Score 1 if all**  **elements are**  **scored as “Y” or “R”** | **Score 1 if all**  **elements are**  **scored as “Y” or “R”** | **Score 1 if all**  **elements are**  **scored as “Y” or “R”** |
|  | **i** | **All predictors are reported**  ***For*** ***development,“all*** ***predictors”refers*** ***to*** ***all*** ***predictors*** ***that*** ***potentially*** ***could*** ***have*** ***been*** ***included*** ***in*** ***the、final’model*** ***(including*** ***those*** ***considered*** ***in*** ***any***  ***univariable*** ***analyses).***  ***For*** ***validation,“all*** ***predictors”means*** ***the*** ***predictors*** ***in*** ***the*** ***model*** ***being*** ***evaluated.*** | **Y** | **Y / N** | **Y / N** | **=Y if D7ai=Y** |
|  | **ii** | **Predictor definitions are clearly presented** | **Y** | **Y / N / R** | **Y / N / R** | **=Y if (D7aii=Y AND V7aii=(Y OR R)) OR (D7aii =(Y OR R)**  **AND V7aii=Y)**  **=R if D7aii=R AND V7aii=R** |

|  | **iii** | **It is clearly described how the predictors were measured** | **Y** | **Y / N / R** | **Y / N / R** | **=Y if (D7aiii=Y AND V7aiii=(Y OR R)) OR (D7aiii =(Y OR R)**  **AND V7aiii=Y)**  **=R if D7aiii=R AND V7aiii=R** |
| --- | --- | --- | --- | --- | --- | --- |
|  | **iv** | **It is clearly described when the predictors were measured** | **Y** | **Y / N / R** | **Y / N / R** | **=Y if (D7aiv=Y AND V7aiv=(Y OR R)) OR (D7aiv=(Y OR R)**  **AND V7aiv=Y)**  **=R if D7aiv=R AND V7aiv=R** |
|  | **7b** | **Report any actions to blind assessment of predictors for the outcome and other predictors.** | **Score 1 if both**  **elements are**  **scored as “Y”** | **Score 1 if both**  **elements are**  **scored as “Y”** | **Score 1 if both**  **elements are**  **scored as “Y”** | **Score 1 if both**  **elements are**  **scored as “Y”** |
|  | **i** | **It is clearly described whether predictor assessments were blinded for outcome *For predictors for which it is clearly a non-issue (e.g. automatic blood pressure measurement, age, sex) and for instances where the predictors were clearly***  ***assessed before outcome assessment, score Yes. For all other predictors an explicit mention is expected.*** | **N** | **Y / N** | **Y / N** | **=Y if D7bi=Y AND V7bi=Y** |
|  | **ii** | **It is clearly described whether predictor assessments were blinded for the other predictors** | **N** | **Y / N** | **Y / N** | **=Y if D7bii=Y AND V7bii=Y** |
| **Sample size** | **8** | **Explain how the study size was arrived at.** | **Score 1 if**  **element is**  **scored as “Y”** | **Score 1 if**  **element is**  **scored as “Y”** | **Score 1 if**  **element is**  **scored as “Y”** | **Score 1 if element is scored as “Y”** |
|  | **i** | **It is explained how the study size was arrived at**  ***Is there any mention of sample size, e.g. whether this was done on statistical grounds or practical/logistical grounds (e.g. an existing study cohort or data set of a RCT was used)?*** | **Y** | **Y / N** | **Y / N** | **=Y if D8i=Y AND V8i=Y** |
| **Missing data** | **9** | **Describe how missing data were handled (e.g., complete-case**  **analysis, single imputation, multiple imputation) with details of any imputation method.** | **Score 1 if all**  **elements are**  **scored as “Y” or “NA”** | **Score 1 if all**  **elements are**  **scored as “Y” or “NA”** | **Score 1 if all**  **elements are**  **scored as “Y” or “NA”** | **Score 1 if all**  **elements are**  **scored as “Y” or “NA”** |

|  | **i** | **The method for handling missing data (predictors and outcome) is mentioned**  ***E.g. Complete case (explicit mention that individuals with missing values have been excluded), single imputation, multiple imputation, mean/median***  ***imputation.***  ***If there is no missing data, there should be an explicit mention that there is no missing data for all predictors and outcome. If so, score Yes.***  ***If it is unclear whether there is missing data (from e.g. the reported methods or results), score No.***  ***If it is clear there is missing data, but the method for handling missing data is unclear, score No.*** | **N** | **Y / N** | **Y / N** | **=Y if D9i=Y AND V9i=Y** |
| --- | --- | --- | --- | --- | --- | --- |
|  | **ii** | **If missing data were imputed, details of the software used are given**  ***When under 9i explicit mentioning of no missing data, complete case analysis or no imputation applied, score Not applicable*** | **N** | **Y / N / NA** | **Y / N / NA** | **=Y if (D9ii=Y AND V9ii=(Y OR NA)) OR (D9ii =(Y OR NA)**  **AND V9ii=Y)**  **=NA if D9ii=NA AND V9ii=NA** |
|  | **iii** | **If missing data were imputed, a description of which variables were included in the imputation procedure is given.**  ***When under 9i explicit mentioning of no missing data, complete case analysis or no imputation applied, score Not applicable*** | **N** | **Y / N / NA** | **Y / N / NA** | **=Y if (D9iii=Y AND V9iii=(Y OR NA)) OR (D9iii =(Y OR NA)**  **AND V9iii=Y)**  **=NA if D9iii=NA AND V9iii=NA** |
|  | **iv** | **If multiple imputation was used, the number of imputations is reported**  ***When under 9i explicit mentioning of no missing data, complete case analysis or no imputation applied, score Not applicable*** | **N** | **Y / N / NA** | **Y / N / NA** | **=Y if (D9iv=Y AND V9iv=(Y OR NA)) OR (D9iv=(Y OR NA)**  **AND V9iv=Y)**  **=NA if D9iv=NA AND V9iv=NA** |
| **Statistical analysis methods** | **10a** | **Describe how predictors were handled in the analyses.** | **Score 1 if all**  **elements are**  **scored as “Y” or “NA”** | **Not applicable** | **Score 1 if all**  **elements are**  **scored as “Y” or “NA”** | **Score 1 if all**  **elements are**  **scored as “Y” or “NA”** |
|  | **i** | **For continuous predictors it is described whether they were modelled as linear, nonlinear (type of transformation specified) or categorized**  ***A general statement is sufficient, no need to describe this for each predictor separately.***  ***If no continuous predictors were reported, score Not applicable.*** | **N** | **Not applicable** | **Y / N / NA** | **=D10ai** |
|  | **ii** | **For categorical or categorized predictors, the cut-points were reported**  ***If no categorical or categorized predictors were reported, score Not applicable.*** | **N** | **Not applicable** | **Y / N / NA** | **= D10aii** |


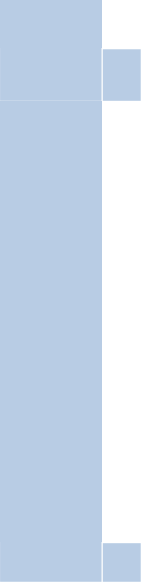

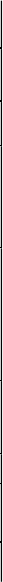


| **iii** | **For categorized predictors the method to choose the cut-points was clearly described**  ***If*** ***no*** ***categorized*** ***predictors,*** ***score*** ***Not*** ***applicable.*** | **N** | **Not applicable** | **Y / N / NA** | **= D10aiii** |
| --- | --- | --- | --- | --- | --- |
| **10b** | **Specify type of model, all model-building procedures (including any predictor selection), and method for internal validation.** | **Score 1 if all**  **elements are**  **scored as “Y” or “NA”** | **Not applicable** | **Score 1 if all**  **elements are**  **scored as “Y” or “NA”** | **Score 1 if all**  **elements are**  **scored as “Y” or “NA”** |
| **i** | **The type of statistical model is reported**  ***E.g.*** ***Logistic,*** ***Cox,*** ***other*** ***regression*** ***model*** ***(e.g.*** ***Weibull,*** ***ordinal),*** ***other*** ***statistical*** ***modelling*** ***(e.g.*** ***neural*** ***network)*** | **Y** | **Not applicable** | **Y / N** | **=D10bi** |
| **ii** | **The approach used for predictor selection before modelling is described**  ***、Before*** ***modelling’means*** ***before*** ***any*** ***univariable*** ***or*** ***multivariable*** ***analysis*** ***of*** ***predictor-outcome*** ***associations.***  ***If*** ***no*** ***predictor*** ***selection*** ***before*** ***modelling*** ***is*** ***done,*** ***score*** ***Not*** ***applicable.***  ***If*** ***it*** ***is*** ***unclear*** ***whether*** ***predictor*** ***selection*** ***before*** ***modelling*** ***is*** ***done,*** ***score*** ***No.*** ***If*** ***it*** ***is*** ***clear*** ***there*** ***was*** ***predictor*** ***selection*** ***before*** ***modelling*** ***but*** ***the*** ***method*** ***was*** ***not*** ***described,*** ***score*** ***No.*** | **Y** | **Not applicable** | **Y / N / NA** | **= D10bii** |
| **iii** | **The approach used for predictor selection during modelling is described**  ***E.g.*** ***Univariable*** ***analysis,*** ***stepwise*** ***selection,*** ***bootstrap,*** ***Lasso.***  ***、During*** ***modelling’includes*** ***both*** ***univariable*** ***or*** ***multivariable*** ***analysis*** ***of*** ***predictor-outcome*** ***associations.***  ***If*** ***no*** ***predictor*** ***selection*** ***during*** ***modelling*** ***is*** ***done*** ***(so-called*** ***full*** ***model*** ***approach),*** ***score*** ***Not*** ***applicable.***  ***If*** ***it*** ***is*** ***unclear*** ***whether*** ***predictor*** ***selection*** ***during*** ***modelling*** ***is*** ***done,*** ***score*** ***No.*** ***If*** ***it*** ***is*** ***clear*** ***there*** ***was*** ***predictor*** ***selection*** ***during*** ***modelling*** ***but*** ***the*** ***method*** ***was*** ***not*** ***described,*** ***score*** ***No.*** | **Y** | **Not applicable** | **Y / N / NA** | **= D10biii** |
| **iv** | **Testing of interaction terms is described**  ***If*** ***it*** ***is*** ***explicitly*** ***mentioned*** ***that*** ***interaction*** ***terms*** ***were*** ***not*** ***addressed*** ***in*** ***the*** ***prediction*** ***model,*** ***score*** ***Yes.***  ***If*** ***interaction*** ***terms*** ***were*** ***included*** ***in*** ***the*** ***prediction*** ***model,*** ***but*** ***the*** ***testing*** ***is*** ***not*** ***described,*** ***score*** ***No.*** | **Y** |  | **Y / N** | **=D10biv** |
| **v** | **Testing of the proportionality of hazards in survival models is described *If*** ***no*** ***proportional*** ***hazard*** ***model*** ***is*** ***used,*** ***score*** ***Not*** ***applicable.*** | **Y** | **Not applicable** | **Y / N / NA** | **=D10bv** |
| **vi** | **Internal validation is reported**  ***E.g.*** ***Bootstrapping,*** ***cross*** ***validation,*** ***split*** ***sample.***  ***If*** ***the*** ***use*** ***of*** ***internal*** ***validation*** ***is*** ***clearly*** ***a*** ***non-issue*** ***(e.g.*** ***in*** ***case*** ***of*** ***very*** ***large*** ***data*** ***sets),*** ***score*** ***Yes.*** ***For*** ***all*** ***other*** ***situations*** ***an*** ***explicit*** ***mention*** ***is*** ***expected.*** | **Y** | **Not applicable** | **Y / N** | **=D10bvi** |
| **10c** | **For validation, describe how the predictions were calculated.** | **Not applicable** | **Score 1 if**  **extraction item**  **is scored as “Y”** | **Score 1 if**  **extraction item**  **is scored as “Y”** | **Score 1 if**  **extraction item is scored as “Y”** |

|  | **i.** | **It is described how predictions for individuals (in the validation set) were obtained from the model being validated**  ***E.g.*** ***Using*** ***the*** ***original*** ***reported*** ***model*** ***coefficients*** ***with*** ***or*** ***without*** ***the*** ***intercept,*** ***and/or*** ***using*** ***updated*** ***or*** ***refitted*** ***model*** ***coefficients,*** ***or*** ***using*** ***a*** ***nomogram,***  ***spreadsheet*** ***or*** ***web*** ***calculator.*** | **Not applicable** | **Y / N** | **Y / N** | **=V10ci** |
| --- | --- | --- | --- | --- | --- | --- |
|  | **10d** | **Specify all measures used to assess model performance and, if relevant, to compare multiple models.2**  ***These*** ***should*** ***be*** ***described*** ***in*** ***the*** ***methods*** ***section*** ***of*** ***the*** ***paper*** ***(item*** ***16*** ***addresses*** ***the*** ***reporting*** ***of*** ***the*** ***results*** ***for*** ***model*** ***performance).*** | **Score 1 if**  **elements 10di**  **and 10dii are**  **scored as “Y”2** | **Score 1 if**  **elements 10di**  **and 10dii are**  **scored as “Y”2** | **Score 1 if all**  **elements are**  **scored as “Y”2** | **Score 1 if**  **elements 10di and 10dii are scored**  **as “Y”2** |
|  | **i** | **Measures for model discrimination are described**  ***E.g.*** ***C-index*** ***/*** ***area*** ***under*** ***the*** ***ROC*** ***curve*** | **Y** | **Y / N** | **Y / N** | **=Y if D10di=Y AND V10di=Y** |
|  | **ii** | **Measures for model calibration are described**  ***E.g.*** ***calibration*** ***plot,*** ***calibration*** ***slope*** ***or*** ***intercept,*** ***calibration*** ***table,*** ***Hosmer*** ***Lemeshow*** ***test,*** ***O/E*** ***ratio.*** | **Y** | **Y / N** | **Y / N** | **=Y if D10dii=Y AND V10dii=Y** |
|  | **iii** | **Other performance measures are described**  ***E.g.*** ***R2,*** ***Brier*** ***score,*** ***predictive*** ***values,*** ***sensitivity,*** ***specificity,*** ***AUC*** ***difference,*** ***decision*** ***curve*** ***analysis,*** ***net*** ***reclassification*** ***improvement,*** ***integrated***  ***discrimination*** ***improvement,*** ***AIC*** | **Y** | **Y / N** | **Y / N** | **=Y if D10diii=Y AND V10diii=Y** |
|  | **10e** | **Describe any model updating (e.g., recalibration) arising from the validation, if done.** | **Not applicable** | **Score 1 if**  **element is**  **scored as “Y”;**  **score *Not***  ***applicable* if**  **element is**  **scored as “NA”** | **Score 1 if**  **element is**  **scored as “Y”;**  **score *Not***  ***applicable* if**  **element is**  **scored as “NA”** | **Score 1 if element is scored as “Y”; score *Not***  ***applicable* if**  **element is scored as “NA”** |
|  | **i** | **A description of model-updating is given**  ***E.g.*** ***Intercept*** ***recalibration,*** ***regression*** ***coefficient*** ***recalibration,*** ***refitting*** ***the*** ***whole*** ***model,*** ***adding*** ***a*** ***new*** ***predictor***  ***If*** ***updating*** ***was*** ***done,*** ***it*** ***should*** ***be*** ***clear*** ***which*** ***updating*** ***method*** ***was*** ***applied*** ***to*** ***score*** ***Yes.***  ***If*** ***it*** ***is*** ***not*** ***explicitly*** ***mentioned*** ***that*** ***updating*** ***was*** ***applied*** ***in*** ***the*** ***study,*** ***score*** ***this*** ***item*** ***as、Not*** ***applicable,.*** | **Not applicable** | **Y / N / NA** | **Y / N / NA** | **=V10ei** |


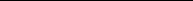


**2 Discrimination and calibration are the two key aspects that characterize the performance of a prediction model and the TRIPOD guideline states that these two measures should be mentioned in every prediction model report. Various other measures of model performance can sometimes be reported (see examples provided at data**

**extraction element 10diii). For reports on D and V and DV, we considered that discrimination and calibration had to be reported to adhere to item 10d. Other overall**

**performance measures such as (R2, Brier score or AIC) were not deemed essential for the scoring of overall adherence in D, V and D+V reports. For reports on the**

**incremental value (IV reports) the reporting of other performance measures, like AUC difference or net reclassification improvement, were considered essential in addition to discrimination and calibration.**

| **Risk groups** | **11** | **Provide details on how risk groups were created, if done.** | **Score 1 if**  **element is**  **scored as “Y”;**  **score *Not***  ***applicable* if**  **element is**  **scored as “NA”** | **Score 1 if**  **element is**  **scored as “Y”;**  **score *Not***  ***applicable* if**  **element is**  **scored as “NA”** | **Score 1 if**  **element is**  **scored as “Y”;**  **score *Not***  ***applicable* if**  **element is**  **scored as “NA”** | **Score 1 if element is scored as “Y”; score *Not***  ***applicable* if**  **element is scored as “NA”** |
| --- | --- | --- | --- | --- | --- | --- |
|  | **i** | **If risk groups were created, risk group boundaries (risk thresholds) are specified *Score this item separately for development and validation if a study includes***  ***both development and validation.***  ***If risk groups were not created, score this item as not applicable.*** | **N** | **Y / N / NA** | **Y / N / NA** | **=Y if (D11i=Y AND V11i=(Y OR NA)) OR (D11i=(Y OR NA)**  **AND V11i=Y)**  **=NA if D11i=NA AND V11i=NA** |
| **Development**  **vs. validation** | **12** | **For validation, identify any differences from the development data in setting, eligibility criteria, outcome and predictors.** | **Not applicable** | **Score 1 if**  **element is**  **scored as “Y”** | **Score 1 if**  **element is**  **scored as “Y” or “NA”** | **Score 1 if element is scored as “Y”** |
|  | **i** | **Differences or similarities in definitions with the development study are described**  ***Mentioning of any differences in all four (setting, eligibility criteria, predictors and outcome) is required to score Yes.***  ***If it is explicitly mentioned that there were no differences in setting, eligibility criteria, predictors and outcomes, score Yes.***  ***For incremental value reports, in case additional predictors are not added to a previously developed prediction model but rather added to conventional***  ***predictors in a newly fitted model, score Not applicable.*** | **Not applicable** | **Y / N** | **Y / N / NA** | **=V12i** |
| **Results** |  |  |  |  |  |  |
| **Participants** | **13a** | **Describe the flow of participants through the study, including the number of participants with and without the outcome and, if**  **applicable, a summary of the follow-up time. A diagram may be helpful.** | **Score 1 if all**  **elements are**  **scored as “Y” or “NA”** | **Score 1 if the**  **elements are**  **scored as “Y” or “NA”** | **Score 1 if all**  **elements are**  **scored as “Y” or “NA”** | **Score 1 if all**  **elements are**  **scored as “Y” or “NA”** |
|  | **i** | **The flow of participants is reported** | **N** | **Y / N** | **Y / N** | **=Y if D13ai=Y AND V13ai=Y** |
|  | **ii** | **The number of participants with and without the outcome are reported *If outcomes are continuous, score Not applicable.*** | **N** | **Y / N / NA** | **Y / N / NA** | **=Y if (D13aii=Y AND V13aii=(Y OR NA)) OR (D13aii =(Y OR NA) AND V13aii=Y)**  **=NA if D13aii=NA AND V13aii=NA** |

|  | **iii** | **A summary of follow-up time is presented**  ***This notably applies to prognosis studies and diagnostic studies with follow-up as diagnostic outcome.***  ***If this is not applicable for an article (i.e. diagnostic study or no follow-up), then score Not applicable.*** | **N** | **Y / N / NA** | **Y / N / NA** | **=Y if (D13aiii=Y**  **AND V13aiii=(Y OR NA)) OR (D13aiii =(Y OR NA) AND**  **V13aiii=Y)**  **=NA if D13aiii=NA AND V13aiii=NA** |
| --- | --- | --- | --- | --- | --- | --- |
|  | **13b** | **Describe the characteristics of the participants (basic demographics, clinical features, available predictors), including the number of**  **participants with missing data for predictors and outcome.** | **Score 1 if all**  **elements are**  **scored as “Y”** | **Score 1 if all**  **elements are**  **scored as “Y”** | **Score 1 if all**  **elements are**  **scored as “Y”** | **Score 1 if all**  **elements are**  **scored as “Y”** |
|  | **i** | **Basic demographics are reported** | **Y** | **Y / N** | **Y / N** | **=Y if D13bi=Y AND V13bi=Y** |
|  | **ii** | **Summary information is provided for all predictors included in the final developed/validated model** | **Y** | **Y / N** | **Y / N** | **=Y if D13bii=Y AND V13bii=Y** |
|  | **iii** | **The number of participants with missing data for predictors is reported** | **N** | **Y / N** | **Y / N** | **=Y if D13biii=Y AND V13biii=Y** |
|  | **iv** | **The number of participants with missing data for the outcome is reported** | **N** | **Y / N** | **Y / N** | **=Y if D13biv=Y AND V13biv=Y** |
|  | **13c** | **For validation, show a comparison with the development data of the distribution of important variables (demographics, predictors and**  **outcome).** | **Not applicable** | **Score 1 if all**  **elements are**  **scored as “Y”** | **Score 1 if all**  **elements are**  **scored as “Y” or “NA”** | **Score 1 if all**  **elements are**  **scored as “Y”** |
|  | **i** | **Demographic characteristics (at least age and gender) of the validation study participants are reported along with those of the original development study *For incremental value reports, in case additional predictors are not added to a previously developed prediction model but rather added to conventional***  ***predictors in a newly fitted model, score Not applicable.*** | **Not applicable** | **Y / N** | **Y / N / NA** | **=V13ci** |
|  | **ii** | **Distributions of predictors in the model of the validation study participants are reported along with those of the original development study**  ***For incremental value reports, in case additional predictors are not added to a previously developed prediction model but rather added to conventional***  ***predictors in a newly fitted model, score Not applicable.*** | **Not applicable** | **Y / N** | **Y / N / NA** | **=V13cii** |
|  | **iii** | **Outcomes of the validation study participants are reported along with those of the original development study**  ***For incremental value reports, in case additional predictors are not added to a previously developed prediction model but rather added to conventional***  ***predictors in a newly fitted model, score Not applicable.*** | **Not applicable** | **Y / N** | **Y / N / NA** | **=V13ciii** |
| **Model**  **development** | **14a** | **Specify the number of participants and outcome events in each analysis.** | **Score 1 if both elements are**  **scored as “Y” or “NA”** | 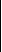**Not applicable** | **Score 1 if both elements are**  **scored as “Y” or “NA”** | **Score 1 if both elements are**  **scored as “Y” or “NA”** |


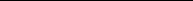


|  | **i** | **The number of participants in each analysis (e.g. in the analysis of each model if more than one model is developed) is specified** | **N** | **Not applicable** | **Y / N** | **=D14ai** |
| --- | --- | --- | --- | --- | --- | --- |
|  | **ii** | **The number of outcome events in each analysis is specified (e.g. in the analysis of each model if more than one model is developed)**  ***If outcomes are continuous, score Not applicable.*** | **N** | **Not applicable** | **Y / N / NA** | **=D14aii** |
|  | **14b** | **If done, report the unadjusted association between each candidate predictor and outcome.** | **Score 1 if**  **element is**  **scored as “Y”;**  **score *Not***  ***applicable* if**  **element is**  **scored as “NA”** | **Not applicable** | **Score 1 if**  **element is**  **scored as “Y”;**  **score *Not***  ***applicable* if**  **element is**  **scored as “NA”** | **Score 1 if element is scored as “Y”; score *Not***  ***applicable* if**  **element is scored as “NA”** |
|  | **i** | **The unadjusted associations between each predictor and outcome are reported *If any univariable analysis is mentioned in the methods but not in the results, score No.***  ***If nothing on univariable analysis (in methods or results) is reported, score this item as Not applicable*** | **Y** | **Not applicable** | **Y / N / NA** | **=D14bi** |
| **Model**  **specification** | **15a** | **Present the full prediction model to allow predictions for individuals (i.e., all regression coefficients, and model intercept or baseline**  **survival at a given time point).** | **Score 1 if both**  **elements are**  **scored as “Y”** | **Not applicable** | **Score 1 if both**  **elements are**  **scored as “Y”** | **Score 1 if both**  **elements are**  **scored as “Y”** |
|  | **i** | **The regression coefficient (or a derivative such as hazard ratio, odds ratio, risk ratio) for each predictor in the model is reported** | **Y** | **Not applicable** | **Y / N** | **=D15ai** |
|  | **ii** | **The intercept or the cumulative baseline hazard (or baseline survival) for at least one time point is reported** | **Y** | **Not applicable** | **Y / N** | **=D15aii** |
|  | **15b** | **Explain how to use the prediction model.** | **Score 1 if**  **element is**  **scored as “Y”** | **Not applicable** | **Score 1 if**  **element is**  **scored as “Y”** | **Score 1 if element is scored as “Y”** |
|  | **i** | **An explanation (e.g. a simplified scoring rule, chart, nomogram of the model,**  **reference to online calculator, or worked example) is provided to explain how to use the model for individualised predictions.** | **Y** | **Not applicable** | **Y / N** | **=D15bi** |
| **Model**  **performance** | **16** | **Report performance measures (with confidence intervals) for the prediction model.3**  ***These should be described in results section of the paper (item 10 addresses the reporting of the methods for model performance).*** | **Score 1 if**  **elements 16i-**  **16iii are scored as “Y”3** | **Score 1 if**  **elements 16i-**  **16iii are scored as “Y”3** | **Score 1 if all**  **elements are**  **scored as “Y”3** | **Score 1 if**  **elements 16i-16iii are scored as “Y” 3** |

**3 See also footnote 2. Discrimination and calibration are the two key aspects that characterize the performance of a prediction model and the TRIPOD guideline states that these two measures should be reported in every prediction model report. Various other measures of model performance can sometimes be reported (see examples**

**provided at data extraction element 16iv). For reports on D and V and D+V, we considered that discrimination and calibration had to be reported to adhere to item 16.**

**Other overall performance measures such as (R2, Brier score or AIC) were not deemed essential for the scoring of overall adherence in D, V and D+V reports. For reports on the incremental value (IV reports) the reporting of other performance measures, like AUC difference or net reclassification improvement, were considered essential in**

**addition to discrimination and calibration.**

|  | **i** | **A discrimination measure is presented**  ***E.g.*** ***C-index*** ***/*** ***area*** ***under*** ***the*** ***ROC*** ***curve*** | **Y** | **Y / N** | **Y / N** | **=Y if D16i=Y AND V16i=Y** |
| --- | --- | --- | --- | --- | --- | --- |
|  | **ii** | **The confidence interval (or standard error) of the discrimination measure is presented** | **Y** | **Y / N** | **Y / N** | **=Y if D16ii=Y AND V16ii=Y** |
|  | **iii** | **Measures for model calibration are described**  ***E.g.*** ***calibration*** ***plot,*** ***calibration*** ***slope*** ***or*** ***intercept,*** ***calibration*** ***table,*** ***Hosmer*** ***Lemeshow*** ***test,*** ***O/E*** ***ratio.*** | **Y** | **Y / N** | **Y / N** | **=Y if D16iii=Y AND V16iii=Y** |
|  | **iv** | **Other model performance measures are presented**  ***E.g.*** ***R2,*** ***Brier*** ***score,*** ***predictive*** ***values,*** ***sensitivity,*** ***specificity,*** ***AUC*** ***difference,*** ***decision*** ***curve*** ***analysis,*** ***net*** ***reclassification*** ***improvement,*** ***integrated***  ***discrimination*** ***improvement,*** ***AIC.*** | **Y** | **Y / N** | **Y / N** | **=Y if D16iv=Y AND V16iv=Y** |
| **Model**  **updating** | **17** | **If done, report the results from any model updating (i.e., model specification, model performance, recalibration).**  ***If*** ***updating*** ***was*** ***not*** ***done,*** ***score*** ***this*** ***TRIPOD*** ***item*** ***as、Not*** ***applicable,.*** | **Not applicable** | **Score 1 if all**  **elements are**  **scored as “Y”** | **Not applicable** | **Score 1 if all**  **elements are**  **scored as “Y”** |
|  | **i** | **The updated regression coefficients for each predictor in the model are reported**  ***If*** ***model*** ***updating*** ***was*** ***described*** ***as、not*** ***needed,,*** ***score*** ***Yes.*** | **Not applicable** | **Y / N** | **Not applicable** | **=V17i** |
|  | **ii** | **The updated intercept or cumulative baseline hazard or baseline survival (for at least one time point) is reported**  ***If*** ***model*** ***updating*** ***was*** ***described*** ***as、not*** ***needed,,*** ***score*** ***Yes.*** | **Not applicable** | **Y / N** | **Not applicable** | **=V17ii** |
|  | **iii** | **The discrimination of the updated model is reported** | **Not applicable** | **Y / N** | **Not applicable** | **=V17iii** |
|  | **iv** | **The confidence interval (or standard error) of the discrimination measure of the updated model is reported** | **Not applicable** | **Y / N** | **Not applicable** | **=V17iv** |
|  | **v** | **The calibration of the updated model is reported** | **Not applicable** | **Y / N** | **Not applicable** | **=V17v** |
| **Discussion** | | | | | | |
| **Limitations** | **18** | **Discuss any limitations of the study (such as nonrepresentative sample, few events per predictor, missing data).** | **Score 1 if**  **element is**  **scored as “Y”** | **Score 1 if**  **element is**  **scored as “Y”** | **Score 1 if**  **element is**  **scored as “Y”** | **Score 1 if element is scored as “Y”** |
|  | **i** | **Limitations of the study are discussed**  ***Stating*** ***any*** ***limitation*** ***is*** ***sufficient.*** | **Y** | **Y / N** | **Y / N** | **=Y if D18i=Y OR V18i=Y** |
| **Interpretation** | **19a** | **For validation, discuss the results with reference to performance in the development data, and any other validation data.** | **Not applicable** | **Score 1 if**  **element is**  **scored as “Y”** | **Score 1 if**  **element is**  **scored as “Y”** | **Score 1 if element is scored as “Y”** |
|  | **i** | **Comparison of results to reported performance in development studies and/or other validation studies is given** | **Not applicable** | **Y / N** | **Y / N** | **=V19ai** |
|  | **19b** | **Give an overall interpretation of the results considering objectives, limitations, results from similar studies and other relevant evidence.** | **Score 1 if**  **element is**  **scored as “Y”** | **Score 1 if**  **element is**  **scored as “Y”** | **Score 1 if**  **element is**  **scored as “Y”** | **Score 1 if element is scored as “Y”** |
|  | **i** | **An overall interpretation of the results is given** | **Y** | **Y / N** | **Y / N** | **=Y if D19bi=Y OR V19bi=Y** |

| **Implications** | **20** | **Discuss the potential clinical use of the model and implications for future research.** | **Score 1 if both**  **elements are**  **scored as “Y”** | **Score 1 if both**  **elements are**  **scored as “Y”** | **Score 1 if both**  **elements are**  **scored as “Y”** | **Score 1 if both**  **elements are**  **scored as “Y”** |
| --- | --- | --- | --- | --- | --- | --- |
|  | **i** | **The potential clinical use is discussed**  ***E.g. an explicit description of the context in which the prediction model is to be used (e.g. to identify high risk groups to help direct treatment, or to triage***  ***patients for referral to subsequent care).*** | **Y** | **Y / N** | **Y / N** | **=Y if D20i=Y OR V20i=Y** |
|  | **ii** | **Implications for future research are discussed**  ***E.g. a description of what the next stage of investigation of the prediction model should be, such as ”We suggest further external validation”.*** | **Y** | **Y / N** | **Y / N** | **=Y if D20ii=Y OR V20ii=Y** |
| **Other information** | | | | | | |
| **Supplementary information** | **21** | **Provide information about the availability of supplementary**  **resources, such as study protocol, web calculator, and data sets.** | **Not included in**  **overall scoring** | **Not included in**  **overall scoring** | **Not included in**  **overall scoring** | **Not included in**  **overall scoring** |
|  | **i** | **Information about supplementary resources is provided** | **N** | **Y / N** | **Y / N** | **=Y if D21i=Y OR V21i=Y** |
| **Funding** | **22** | **Give the source of funding and the role of the funders for the present study.** | **Score 1 if both**  **elements are**  **scored as “Y”** | **Score 1 if both**  **elements are**  **scored as “Y”** | **Score 1 if both**  **elements are**  **scored as “Y”** | **Score 1 if both**  **elements are**  **scored as “Y”** |
|  | **i** | **The source of funding is reported or there is explicit mention that there was no external funding involved** | **Y** | **Y / N** | **Y / N** | **=Y if D22i=Y OR V22i=Y** |
|  | **ii** | **The role of funders is reported or there is explicit mention that there was no external funding** | **Y** | **Y / N** | **Y / N** | **=Y if D22ii=Y OR V22ii=Y** |
