# Supplementary material for: Systematic review of risk prediction models for sepsis-associated brain dysfunction
Source: Front Neurol. 2026 Feb 27;17:1653460. doi: 10.3389/fneur.2026.1653460 (PMC12982069; doi:10.3389/fneur.2026.1653460)
Supplement: Supplementary file 1 [file Data_Sheet_1.zip › Dataset Appendix.docx]

| Database | Source Institution | Time Span | Sample Size | Core Data Characteristics | Key Variables Relevant  to This Study |
| --- | --- | --- | --- | --- | --- |
| MIMIC-III | Beth Israel Deaconess Medical Center (BIDMC), USA | 2001-2012 | 53,423 adult patients+ 7,870 neonatal patients;58,976 hospital admissions, 61,532 ICU stays | Structured data: Vital signs (hourly intervals), laboratory results, medication records (name, dosage, route), ICD-9 diagnoses/procedures, fluid balance, length of stay | Age, temperature, mechanical ventilation, SOFA score, APACHE II score, sodium, S100B, lactate |
|  |  |  |  | Unstructured data: Nursing notes, imaging reports |  |
|  |  |  |  | 26 relational tables linked by SUBJECT_ID, HADM_ID, ICUSTAY_ID. |  |
| MIMIC-IV | Beth Israel Deaconess Medical Center (BIDMC), USA | 2008-2019 | >190,000 patients;450,000 hospital admissions, >65,000 ICU stays, >200,000 ED visits | Modular structure: Hosp (laboratory/microbiology/medications/ICD-10 codes), ICU (hourly vital signs/fluid balance), ED (triage/treatment), CXR (chest X-ray images/reports), Note (deidentified clinical notes) | Age, vasopressor use, mechanical ventilation, SOFA score, APACHE II score, sodium, albumin |
|  |  |  |  | Combined structured and unstructured data |  |
|  |  |  |  | Improved data traceability with source database |  |
| eICU-CRD | 208 hospitals in the continental USA (participating in Philips Healthcare eICU Telehealth Program) | 2014-2015 | 139,367 patients;139,367 ICU admissions | Multicenter structured data: Vital signs (5-minute intervals, 16 indicators), laboratory tests (160 standard items), medication prescriptions, treatment records (mechanical ventilation, dialysis), ICD-9/10 diagnoses;- Identifiers (HOSPITALID, UNIQUE_PID, PATIENTUNITSTAYID) for data linkage | Mechanical ventilation, vasopressor use, SOFA score, age, temperature, ICU length of stay |
|  |  |  |  | Time stamps recorded as offsets from ICU admission. |  |
| Chinese Single-Center Cohorts | Shanghai, Jiangsu, Ningxia, Guizhou, Xinjiang | 2019-2024 | 8 included studies^[16,19-25]^; 1,900 patients | Single-center retrospective/prospective data: Demographics (age, gender), clinical indicators (SOFA/APACHE II scores), treatment records (mechanical ventilation, vasopressors), laboratory results | Age, APACHE II score, SOFA score, mechanical ventilation, vasopressor use, sodium, S100B |

Note:MIMIC-III, Medical Information Mart for Intensive Care III; MIMIC-IV, Medical Information Mart for Intensive Care IV;eICU-CRD,eICU Collaborative Research Database.
